# Testis formation in XX individuals resulting from novel pathogenic variants in Wilms’ tumor 1 (*WT1*) gene

**DOI:** 10.1073/pnas.1921676117

**Published:** 2020-06-03

**Authors:** Caroline Eozenou, Nitzan Gonen, Maria Sol Touzon, Anne Jorgensen, Svetlana A. Yatsenko, Leila Fusee, Alaa K. Kamel, Balazs Gellen, Gabriela Guercio, Priti Singh, Selma Witchel, Andrea J. Berman, Rana Mainpal, Mehdi Totonchi, Anahita Mohseni Meybodi, Masomeh Askari, Tiphanie Merel-Chali, Joelle Bignon-Topalovic, Roberta Migale, Mariana Costanzo, Roxana Marino, Pablo Ramirez, Natalia Perez Garrido, Esperanza Berensztein, Mona K. Mekkawy, John C. Schimenti, Rita Bertalan, Inas Mazen, Ken McElreavey, Alicia Belgorosky, Robin Lovell-Badge, Aleksandar Rajkovic, Anu Bashamboo

**Affiliations:** ^a^Human Developmental Genetics Unit, Institut Pasteur, 75724 Paris, France;; ^b^Laboratory of Stem Cell Biology and Developmental Genetics, The Francis Crick Institute, NW1 1AT London, United Kingdom;; ^c^The Mina and Everard Goodman Faculty of Life Sciences, Bar-Ilan University, 5290002 Ramat Gan, Israel;; ^d^Endocrinology Department, Research Unit Garrahan Consejo Nacional de Investigaciones Cientificas y Tecnologicas, Hospital de Pediatría Garrahan, Ciudad Autonoma de Buenos Aires 1245, Argentina;; ^e^Department of Growth and Reproduction, Copenhagen University Hospital (Rigshospitalet), 2100 Copenhagen, Denmark;; ^f^Department of Obstetrics, Gynecology & Reproductive Sciences, Magee-Womens Research Institute, University of Pittsburgh, Pittsburgh, PA 15213;; ^g^Department of Pathology, Magee-Womens Research Institute, University of Pittsburgh, Pittsburgh, PA 15213;; ^h^Department of Human Genetics, Magee-Womens Research Institute, University of Pittsburgh, Pittsburgh, PA 15213;; ^i^Division of Human Genetics and Genome Research, National Research Centre, Dokki, Cairo, 12622, Egypt;; ^j^Department of Paediatrics, University of Szeged, 6725 Szeged, Hungary;; ^k^Department of Biomedical Sciences, Cornell University, Ithaca, NY 14853;; ^l^Department of Pediatrics, University of Pittsburgh Medical Center, Children’s Hospital of Pittsburgh, University of Pittsburgh, Pittsburgh, PA 15213;; ^m^Department of Biological Sciences, University of Pittsburgh, Pittsburgh, PA 15213;; ^n^Department of Genetics, Reproductive Biomedicine Research Center, Royan Institute for Reproductive Biomedicine, Academic Center for Education, Culture and Research, 8158968433 Tehran, Iran;; ^o^Department of Stem Cells and Developmental Biology, Cell Science Research Center, Royan Institute for Stem Cell Biology and Technology, Academic Center for Education, Culture and Research, 8158968433 Tehran, Iran;; ^p^1st Department of Pediatrics, Semmelweis University, 1083 Budapest, Hungary;; ^q^Department of Pathology, Obstetrics, Gynecology and Reproductive Sciences, University of California, San Francisco, CA 94158

**Keywords:** 46,XX TDSD/OTDSD, WT1, sex determination, β-CATENIN, organogenesis

## Abstract

Sex determination in mammals is governed by antagonistic interactions of two genetic pathways, imbalance in which may lead to disorders/differences of sex development (DSD) in human. Among 46,XX individuals with testicular DSD (TDSD) or ovotesticular DSD (OTDSD), testicular tissue is present in the gonad. Although the testis-determining gene *SRY* is present in many cases, the etiology is unknown in most *SRY*-negative patients. We performed exome sequencing on 78 individuals with 46,XX TDSD/OTDSD of unknown genetic etiology and identified seven (8.97%) with heterozygous variants affecting the fourth zinc finger (ZF4) of Wilms’ tumor 1 (WT1) (p.Ser478Thrfs*17, p.Pro481Leufs*15, p.Lys491Glu, p.Arg495Gln [x3], p.Arg495Gly). The variants were de novo in six families (*P* = 4.4 × 10^−6^), and the incidence of WT1 variants in 46,XX DSD is enriched compared to control populations (*P* < 1.8 × 10^−4^). The introduction of ZF4 mutants into a human granulosa cell line resulted in up-regulation of endogenous Sertoli cell transcripts and *Wt1*^*Arg495Gly/Arg495Gly*^ XX mice display masculinization of the fetal gonads. The phenotype could be explained by the ability of the mutated proteins to physically interact with and sequester a key pro-ovary factor β-CATENIN, which may lead to up-regulation of testis-specific pathway. Our data show that unlike previous association of WT1 and 46,XY DSD, ZF4 variants of WT1 are a relatively common cause of 46,XX TDSD/OTDSD. This expands the spectrum of phenotypes associated with WT1 variants and shows that the WT1 protein affecting ZF4 can function as a protestis factor in an XX chromosomal context.

## Significance

Sex development involves a precise spatiotemporal expression and interactions of numerous genetic factors, including the *WT1* (Wilms tumor 1) gene. Complete and partial loss-of-function *WT1* variants are associated with 46,XY disorders/differences of sex development (DSD). Some 46,XX individuals develop testis in absence of the testis-determining gene *SRY*. We describe a genotype/phenotype association where variants impacting the C-terminal zinc finger (ZF4) of WT1 cause testis development in 46,XX individuals. XX mice carrying a pathogenic variant of ZF4 display masculinization of the fetal gonads. Testis formation may be due to inappropriate interaction between the mutated WT1 and an essential ovarian determinant β-CATENIN. These data show that variants affecting a specific domain of a developmental transcription factor can switch organ fate.

In mammals, the initial events of sex determination are genetically determined (XX female or XY male). In humans, the early embryonic gonad remains “bipotential” until around gestational week (GW) 6. The product of the testis-determining gene *SRY* on the Y chromosome is the molecular switch that functions by up-regulating *SOXE* gene expression, notably *SOX9*, thereby initiating a series of events leading to testis formation as well as repression of pro-ovarian development (1–4). In the absence of *SRY*, several ovary-specific pathways become dominant or are activated, including WNT4/RSPO1, which stabilizes the downstream effector β-CATENIN, an independent pathway involving FOXL2 and a recently described pathway involving RUNX1 (2–4). The extent to which these networks interact is unknown. Pathogenic variants or anomalous regulation of components of the sex-determining/maintaining networks give rise to DSD (disorders/differences in sex development). DSDs are a complex group of rare congenital conditions where chromosomal, gonadal, or anatomical sex is discordant and include a wide range of phenotypes affecting the endocrine and reproductive systems (5–7). DSDs consist of a continuum of phenotypes ranging from complete sex reversal to that of a newborn with atypical genitalia or postpubertal individuals with primary amenorrhea. The complexity of the DSD phenotypes is reflected by the variation in the genetic etiologies. Sex chromosome mosaicism (45,X/46,XY) and 46,XY DSD (gonadal dysgenesis, disorders of androgen synthesis and action) are relatively common forms of DSD. Most individuals with 46,XX DSD have congenital adrenal hyperplasia (CAH) secondary to 21 α-hydroxylase deficiency (Mendelian Inheritance in Man [MIM] 201910). Importantly, a subset of individuals with a 46,XX karyotype present with testicular DSD (TDSD) or ovotesticular DSD (OTDSD) (5–7). Individuals with TDSD are males with small and azoospermic testis (8) and a normal male habitus. 46,XX OTDSD refers to individuals that have both ovarian and testicular tissue in the gonads, usually ovotestes but less commonly a testis (or ovotestis) on one side and an ovary on the other (9). The external genitalia are usually ambiguous or feminine, with the degree of masculinization broadly correlating with the amount of testicular tissue present. In both TDSD and OTDSD, the histological examination of the gonads shows distinct tubular structures in the testis-like tissue and the presence of follicles in the ovarian-like tissue, in the case of OTDSD.

Our understanding of the molecular causes of TDSD/OTDSD is incomplete. Most nonsyndromic children with these pathologies will carry the *SRY* gene usually on the X chromosome. Others have chromosomal rearrangements resulting in the dysregulation of expression of SRY-related *SOX* genes (e.g., *SOX9, SOX3, SOX10*) (10–13). Despite the absence of *SRY*, these *SOX* genes act as testis-promoting factors, when expressed ectopically in cells of the bipotential supporting cell lineage. Other syndromic forms of TDSD/OTDSD have loss-of-function (LOF) variants in genes in the WNT signaling pathway, which are expressed in the developing ovary and actively repress testis development (e.g., *RSPO1, WNT4*) (14, 15). More recently, LOF variants in the *NR2F2* gene encoding the transcriptional repressor COUP-TFII cause a syndromic form of TDSD/OTDSD and a specific amino acid change in the NR5A1 protein is associated with nonsyndromic TDSD/OTDSD (16, 17). Nevertheless, despite these advances, most cases of *SRY*-negative TDSD/OTDSD remain unexplained at a molecular level.

The human Wilms’ tumor 1 (*WT1*) gene encodes a transcription factor containing four zinc finger motif DNA-binding domains at the C terminus and a proline/glutamine-rich domain at the N terminus. All WT1 isoforms include the four zinc fingers. WT1 plays an essential role in the normal development of the urogenital system, heart, and diaphragm. Haploinsufficiency of *WT1* is linked with the WAGR syndrome (Wilms’ tumor, aniridia, genitourinary anomalies and retardation; MIM 194072), whereas specific pathogenic variants cause two rare autosomal dominant diseases; Frasier syndrome (MIM 136680; splice site variants) and Denys–Drash syndrome (MIM 194080; variants in exon 8 or 9, encoding zinc fingers 2 and 3 [ZF2 and ZF3]). In both conditions, 46,XY children are undervirilized. Affected children typically present with ambiguous or female external genitalia, gonadal dysgenesis, renal anomalies, and renal disease (18–20). Rare genetic variants in *WT1* have also been reported in association with Meacham syndrome (MIM 608978), characterized by 46,XY gonadal dysgenesis, congenital diaphragmatic hernia, and congenital heart disease (21). *WT1* variants are seen in 46,XX girls in association with either apparently normal functioning ovaries, primary ovarian insufficiency, or streak gonads in association with nephrotic syndrome due to either diffuse mesangial sclerosis or focal segmental glomerulosclerosis (nephrotic syndrome type 4; MIM 256370) (22, 23).

Here, we identified de novo pathogenic variants impacting the highly conserved ZF4 of WT1 associated with testis development in a cohort of 46,XX *SRY*-negative TDSD/OTDSD. In vitro transient transactivation assays show that WT1 ZF4 variants have aberrant biological activity as compared to the WT protein. When introduced into a human granulosa cell line, these variants result in up-regulation of Sertoli-specific transcripts. Moreover, a specific genome edit to create an identical amino acid change into ZF4 of mouse WT1 results in masculinization of the embryonic XX gonad. Mutant WT1 ZF4 proteins were observed to physically interact with and sequester a key pro-ovarian and antitestis factor, β-CATENIN, suggesting that these are gain-of-function variants. In this model, testis formation is induced by destabilization of the balance between pro-ovary and protestis gene regulatory networks that operate in the early gonad, favoring the latter. As a result, the variants specifically affecting ZF4 of WT1 can initiate testis formation in XX individuals and are therefore a cause of 46,XX TDSD/OTDSD.

## Methods

We studied 78 children with 46,XX TDSD/OTDSD of unknown etiology (Table 1). All patients were screened for variants in genes known to cause DSD either by analysis of exome datasets or by direct Sanger sequencing of candidate genes. Details regarding extended clinical data, variant screening, in silico protein modeling of WT1 mutant proteins, expression profiling of human fetal tissue, in vitro assays, and generation of mutant mice are described in *SI Appendix* available with the full text of this article.

**Table 1.** Phenotypes, genotypes, and investigation of gonadal histology in eight individuals with pathogenic variants in the *WT1* gene

Variable	Patient 1	Patient 2	Patient 3	Patient 4	Patient 5a	Patient 5b	Patient 6	Patient 7
Sex of rearing	Male	Female	Male	Female	Female	Male	Male	Female
Karyotype	XX	XX	XX	XX	XX	XY	XX	XX
Age at presentation	5 y	2.5 y old	Birth	Birth	Birth	Birth	32 y	Birth
Diagnosis	Testicular DSD	Testicular DSD	Testicular DSD	Ovotesticular DSD	Ovotesticular DSD	Meacham Syndrome	Testicular DSD	Suspected Ovotesticular DSD
Clinical phenotype	Atypical genitalia (Prader IV), microcephaly (-4.5 SD)	Atypical genitalia (Prader IV); mullerian structures	Atypical genitalia (Prader IV); mullerian structures	Atypical genitalia (Prader IV); normal uterus	Atypical genitalia (Prader IV)	Male external genitalia, anorchia, diaphragmatic hernia	Male external genitalia. Testis size of 8 cc. Infertile	Diaphragmatic hernia, single perineal opening, clitoromegaly (Prader IV)
Gonadal position	Nonpalpable	Nonpalpable	Nonpalpable	Nonpalpable.	Nonpalpable	Nonpalpable	Palpable	Nonpalpable
Gonad and gonadal histology	Bilateral dysgenetic	Bilateral dysgenetic	Bilateral dysgenetic	Bilateral ovotestis	Bilateral ovotesis	Right, macroscopically a rudimentary testis in the abdomen	Bilateral testis	Pelvic ultrasound showed bicornuate uterus, and apparently normal ovaries.
	Testicular tissue with fibrosed and hyalinized tubules, interstitial cellular hyperplasia. No germ cells	Testicular parenchyma	Testicular parenchyma	Gonadoblastoma and dysgerminoma	Seminiferous tubules with Sertoli and Leydig cells and areas with pregranulosa cells and primary follicles. No germ cells.	Left, no testicular tissue only pieces of funiculus spermaticus and epididymis tissue	Gonadal Histology not available	No gonadal biopsies were performed.
Ancestry	Egyptian	Caucasian	Caucasian	Caucasian	Hungarian	Hungarian	Iranian	Caucasian
WT1 mutation inheritance	c.1483C > G p:Arg495Gly de novo	c.1442_1449delCCTTCAGC p.Pro481Leufs*15 de novo	c.1484G > A p.Arg495Gln de novo	c.1484G > A p.Arg495Gln de novo	c.1484G > A p.Arg495Gln de novo	c.1484G > A p.Arg495Gln de novo	c.1471A > G p.Lys491Glu Unknown	c.1433–1G > A p.Ser478Thrfs*17 de novo

All patients with 46,XX DSD met the revised criteria of the Pediatric Endocrine Society/European Society for Pediatric Endocrinology. We obtained written informed consent from all patients and family members who participated in the study. This study was approved by the local French ethical committee (2014/18NICB; registration no. IRB00003835) and Independent Ethical Committee at Hospital de Pediatria Garrahan (2016/971). Consent to genetic testing was obtained from adult probands or from the parents when the patient was under 18 y. For analysis of human gonad tissue, the Danish regional ethics committee approved this study (permit no. H-1-2012-007), and women gave their informed written and oral consent. None of the terminations were for reasons of fetal abnormality, and all fetuses appeared morphologically normal. All procedures involving animals and their care conformed to institutional, state, and national guidelines or laws. This study was approved by the Animal Ethics Committee and by the UK Home Office (PPL 70/8560).

## Results

### Clinical Phenotypes.

**Fig. 1.**
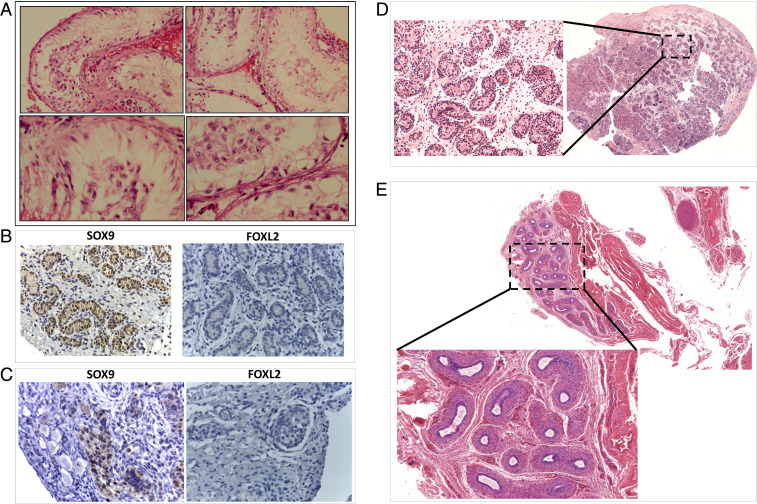
Histology of gonads from patients showing testicular or ovotesticular XX DSD. (*A*) Gonadal histology of Patient 1 showing bilateral dysgenetic testis. Sections of right and left gonads show fibrosed and hyalinized tubules as well as tubules lined by Sertoli cells only, with no evidence of germ cells, along with marked interstitial cellul hyperplasia. There is no evidence of ovarian tissue or malignancy. Section of the uterus shows a muscular uterine wall lined by inactive endometrium. (*B*) Immunohistochemical (IHC) analysis of both gonads from Patient 3 showed testis with signs of dysgenesis: immature seminiferous cords infiltrating tunica albuginea with few germ cells and vacuolated Sertoli cells. No Leydig cells were identified. There were some oversized germ cells centrally located, resembling gonocytes, in the left gonad. (*C*, *Left*) Gonad of Patient 4 revealed dysgenetic seminiferous tubules in a cellular ovarian-like stroma. Germ cells with dysplastic features, outside and inside the seminiferous tubules were found. Incipient gonadoblastoma-like structures were identified, showing SOX9 staining. The right gonad showed dysgenetic seminiferous cords surrounded by fibrous interstitium with vacuolated Sertoli cells and apoptotic germ cells. A SOX9-positive gonadoblastoma was also identified. A secondary follicle displaying FOXL2 positivity was found. Both gonads were classified as ovotestes considering the presence of ovarian follicles coexisting with the testicular parenchyma. (*D*) The histological section of the gonad of Patient 5a shows immature testicular tissue with Sertoli cells. Leydig cells were not detected. (*E*) Histology of the left side internal genitalia of Patient 5b at the age of 1.8 y shows no testicular tissue. Only the funiculus spermaticus and epididymis tissue were seen (hematoxylin-eosin staining).

All individuals with TDSD/OTDSD were 46,XX with varying degrees of virilization (Table 1). Patients 1, 2, and 3 presented with 46,XX TDSD with their external genitalia ranging from typical male habitus to ambiguous. Patient 4 presented with 46,XX OTDSD. Patients 5a and 5b are a sib pair with 46,XX OTDSD and Meacham syndrome with 46,XY gonadal dysgenesis, respectively. Patient 6 has typical male habitus and presented with 46,XX TDSD. Patient 7 presented with congenital diaphragmatic hernia, masculinized external genitalia, and suspected 46,XX OTDSD. Diagnostic criteria of all of the patients are summarized in Table 1, and extended clinical information is in *SI Appendix*. Gonadal histology for Patients 1, 3, 4, 5a, and 5b (Fig. 1), and kidney ultrasound for Patient 7 (*SI Appendix*, Fig. S1) was available.

### Pathogenic Variants in ZF4 of WT1 Are Associated with 46,XX TDSD/OTDSD and 46,XY Meacham Syndrome.

**Fig. 2.**
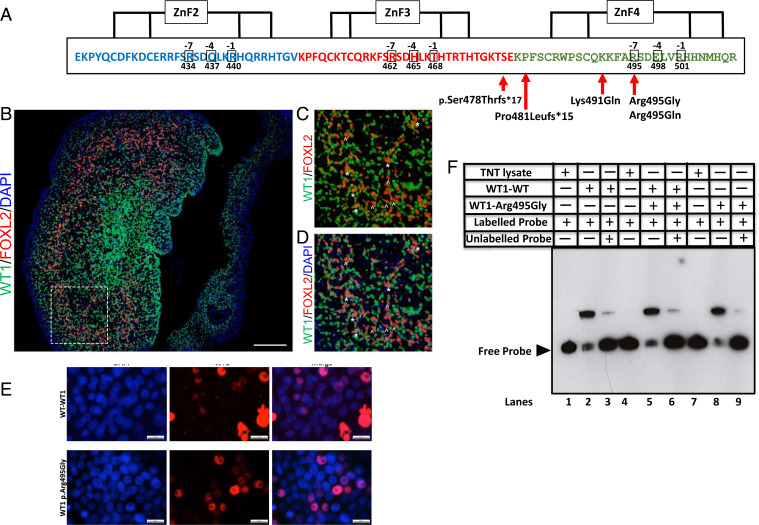
Mutations in *WT1* associated with 46,XX TDSD/OTDSD, cellular localization of WT1, and DNA-binding capacity of WT1p.Arg495Gly. (*A*) The zinc finger domain structure of the human WT1 protein is shown indicating the position of the mutations associated with TDSD/OTDSD. WT1 binds to DNA motifs with the sequence 5′-GCG(T/G)GGGCG-3′ via its C2H2-type zinc fingers. Starting from the N terminus, the second zinc finger binds to the 3′GCG motif, the third zinc finger interacts with the central TGG motif, and the fourth zinc finger binds to the 5′-GCG motif. (*B*–*D*) Immunofluorescence showing protein localization of WT1 (green) and granulosa cell marker FOXL2 (red) in human fetal ovaries from GW 9 + 5. WT1 expression is observed in both FOXL2 negative and positive stromal cell populations at this relatively early developmental time-point. Dashed lines in *B* indicates the position of the expanded view. Nuclei are stained with DAPI. In *C* and *D*, asterisk (*) marks cells coexpressing WT1 and FOXL2 and caret (^) marks cells only expressing FOXL2. (Scale bar: 100 μm.) (*E*) Human embryonic kidney HEK293-T cells were transfected with WT1-WT or WT1p.Arg495Gly expression vectors. The subcellular localization of the proteins was studied by Immunocytochemistry using anti-WT1 antibody. Both WT1-WT and WT1p.Arg495Gly localized to the nucleus. (Scale bar, 20 μm.) (*F*) DNA binding of WT1. EMSA on WT1-WT and WT1p.Arg495Gly was performed using digoxigenin (DIG)-labeled probe corresponding to the WT1 response element with each protein alone as well as a combination of both the WT and the mutant protein. TNT lysate (lanes 1, 4, 7) and competition with 100-fold unlabeled primer (lanes 3, 6, 9) were used to determine the specificity of binding. Both the WT and mutant WT1 bind to the minimum consensus sequence alone (lanes 2, 8) or as a heterodimer (lane 5).

We identified seven families from a cohort of 78 unrelated 46,XX TDSD/OTD individuals (8.97%) carrying five different novel variants in WT1 (p.Arg495Gly [Patient 1]; p.Pro481Leufs*15 [Patient 2]; p.Arg495Gln [Patients 3, 4, 5a, 5b]; Lys491Glu [Patient 6]; p.Ser478Thrfs*17 [Patient 7]; Table 1). In Family 5, an affected XY sib presented with Meacham syndrome (Patient 5b). All of the variants clustered in a region that is highly conserved in vertebrate species, which codes for the ZF4 of the protein (Fig. 2*A* and *SI Appendix*, Fig. S2). None of the *WT1* variants were identified in more than 400 known fertile individuals (in-house controls) and were also absent from public databases (ExAC database http://exac.broadinstitute.org and gnomAD database https://gnomad.broadinstitute.org). Fisher’s Exact test (two-tailed) on the frequency of LOF and nonsynonymous variants associated with 46,XX DSD observed in our cohort, compared with rare (Minor Allelic Frequency [MAF] < 0.01%) LOF and nonsynonymous variants in the *WT1* gene from control individuals, shows a highly significant enrichment of these *WT1* variants in the patient cohort (*P* < 1.8 × 10^−4^). In six families the mode of inheritance was de novo, while it is unknown in one remaining case (Patient 6), because DNA from the parents was unavailable for study. Compared to the frequency of de novo nonsynonymous/LOF variants in control individuals (0/522, http://denovo-db.gs.washington.edu/denovo-db), the DSD cohort is highly enriched for de novo variants (*P* = 4.1 × 10^−6^). Moreover, all of the families were unrelated and came from geographically distinct regions, excluding a founder effect for rare variants. All 46,XX children were *SRY-*negative, and the exome data analysis did not identify any other likely pathogenic variants in known sex determination/DSD genes. An analysis of ClinVar (https://www.ncbi.nlm.nih.gov/clinvar) for pathogenic variants involving the ZF4 of WT1 indicated several missense variants (p.Pro487Ser, Variation ID: 543123; p.Ser488Asn [x2], Variation ID: 578607; p.Lys491Arg, Variation ID: 655847; p.His505Gln, Variation ID: 664113) in association with the typical clinical features of WT1 pathogenic variants (Denys–Drash syndrome, Wilms tumor, aniridia, genitourinary anomalies, mental retardation syndrome, Frasier syndrome). The presence of pathogenic variants among affected individuals in our study and those submitted to the ClinVar database, and the absence of similar changes in public databases, underscores the functional importance of the ZF4 of WT1.

### WT1 Protein Is Expressed in Stromal and Granulosa Cells of the Early Human Embryonic Ovary.

To determine where WT1 is expressed in the developing human ovary, immunohistochemical studies were performed using GW9+5 fetal tissue. WT1 expression is observed in both the stromal cells and a subpopulation of the FOXL2-positive granulosa cells. (Fig. 2 *B*–*D*).

### WT1p.Arg495Gly Alters the Biological Activity of the Protein.

**Fig. 3.**
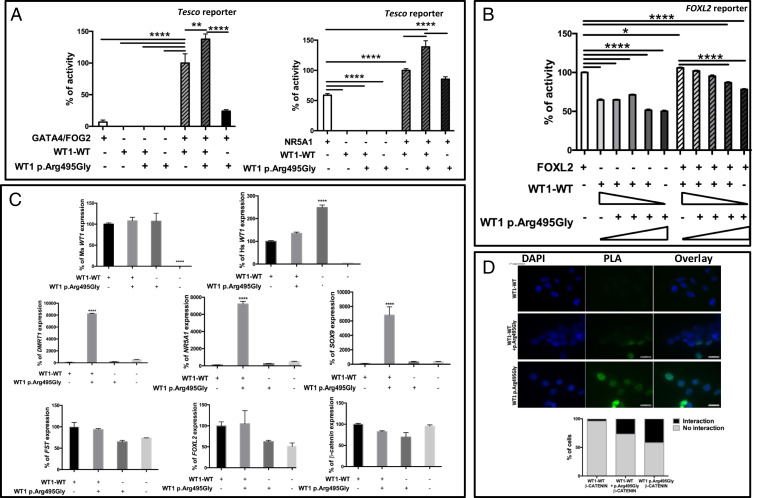
In vitro assays for biological activity of WT1p.Arg495Gly. (*A*) The transcriptional activities of WT1-WT and WT1p.Arg495Gly were studied using the mouse *Sox9 Tesco* enhancer as a reporter, following transfection in HEK293-T cells. All data were standardized for Renilla activity. The data shown represent the mean ± SEM of minimum three independent experiments, each of which was performed at least in quadruplicate. The reporter constructs were transfected into HEK293-T cells with either the WT1-WT or WT1p.Arg495Gly expression vector. The results are expressed as relative percentage of synergistic activation by WT1-WT and GATA4/FOG2 (100%; *Left*) or WT1-WT and NR5A1 (100%; *Right*). The WT1p.Arg495Gly together with WT1-WT shows an enhanced synergistic activation of *Sox9 Tesco* enhancer, both with NR5A1 and GATA4/FOG2, as compared to that by WT or WT1p.Arg495Gly alone. The statistically significant differences (*P* ≤ 0.05) are marked by an asterisk (*) above the data points in the graphs. (*B*) The transcriptional activities of WT1-WT and WT1p.Arg495Gly were studied using the goat *FOXL2* promoter as reporter, following transfection in HEK293-T cells. All data were standardized for Renilla activity. The data shown represent the mean ± SEM of a minimum of three independent experiments, each of which was performed at least in quadruplicate. The reporter constructs were transfected into HEK293-T cells with increasing doses of either the WT1-WT or WT1p.Arg495Gly expression vector with or without *FOXL2* expression vector. The results are expressed as relative percentage activation by *FOXL2* alone (100%). The WT1p.Arg495Gly mutant shows a dose-dependent repression of synergistic activation of *FOXL2* promoter by WT1-WT and FOXL2. (*C*) Human granulosa KGN-1 cells were transfected with plasmids encoding mouse WT1-WT and WT1p.Arg495Gly expression vectors together or each on its own. After transfections, the levels of exogenous (mouse) and endogenous (human) WT1 and Sertoli-specific (*SOX9, NR5A1*, *DMRT1*) and granulosa-specific (*FOXL2,* β*-CATENIN, FST*) transcripts were measured by qRT-PCR. Data are presented as mean 2-ΔΔCt values, normalized to the housekeeping gene 18S rRNA normalizer gene (RPL19). When transfected together, the WT1-WT and WT1p.Arg495Gly result in multifold induction of endogenous protestis transcripts, whereas the levels of pro-ovary transcripts remain unchanged. (*D*) Plasmids encoding β-CATENIN and WT1-WT or WT1p.Arg495Gly were transiently expressed for 48 h in HEK293-T cells. Protein–protein interaction of WT1-WT and WT1p.Arg495Gly with β-CATENIN was analyzed using the Duolink PLA. Nuclei are stained with DAPI (blue) and Duolink signal representing interaction between the proteins is shown in green. (Scale bars, 20 μm.) In contrast to the WT, WT1p.Arg495Gly protein shows a higher degree of interaction with βCATENIN protein. For each condition, at least 300 individual cells were counted and the percentage of cells showing interaction were calculated.

The in silico protein modeling predicted the variants to have severely deleterious impact on the structural stability of the ZF4 of WT1 (*SI Appendix*, Fig. S3). Using WT1p.Arg495Gly variant as a proof of concept, we investigated the functional consequences of mutating ZF4 of WT1 as well as the possible disease mechanisms using in vitro and in situ assays. The subcellular localization of both the WT and mutant WT1 proteins was determined by transient transfection assays in human embryonic kidney HEK293-T cells. We observe a strong nuclear localization for both mutant and WT proteins (Fig. 2*E*). The ZF4 has been reported to stabilize the DNA binding of ZF2 and ZF3 (24). Therefore, we investigated the ability of the mutant protein to bind to WT1 target consensus sequence by electrophoretic mobility shift assay (EMSA), and we found that the mutant protein maintains DNA-binding activity (Fig. 2*F*). We hypothesized that testis development associated with the WT1p.Arg495Gly variant may be due to inappropriate activation of testis-specific pathways in the ovary, either by direct activation of protestis genes or indirectly by disrupting pathways that normally oppose testis formation. Using reporter assays in transiently transfected HEK293-T cells, the WT1p.Arg495Gly shows a statistically significant increase in the activation of the testis-specific enhancer of *Sox9* (*Tesco*) in conjunction with the cofactors GATA4/FOG2 and NR5A1 (Fig. 3*A*). WT1p.Arg495Gly also shows a dose-dependent reduction in activation of the ovary-specific *FOXL2* promoter (Fig. 3*B*). It has previously been suggested that WT1 and FOXL2 proteins may interact (25), and we found that the mutated WT1 protein showed binding with the FOXL2 protein similar to the wild type (*SI Appendix*, Fig. S4). Transient transfection of WT1p.Arg495Gly into the human granulosa KGN-1 cell line results in a multifold up-regulation of endogenous transcripts associated with Sertoli cell formation (*SOX9*, *NR5A1*, *DMRT1*), whereas the expression levels of endogenous granulosa transcripts (*FOXL2*, β*CATENIN*, *FST*) remains unchanged (Fig. 3*C*). We observe up-regulation of endogenous *SOX9* expression after transfection of KGN-1 cells with two other variants, which affect ZF4 (WT1p.Arg495* and WT1p.Lys491Glu). The levels of endogenous *FOXL2* remain unchanged (*SI Appendix*, Fig. S5). A variant in ZF3 (WT1p.Gln437Lys) has been reported in a pair of 46,XX monozygotic twins with renal disease but no evidence of virilization (22). Notably, *SOX9* expression was not up-regulated following transfection with this variant, which is consistent with the clinical phenotype.

The determination of ovary or testis from the bipotential gonad during mammalian development is governed by mutually antagonistic regulatory networks involving β-CATENIN (ovary) and SOX9 (testis). Using the proximity ligation assay (PLA), we observe that WT1p.Arg495Gly shows significantly increased binding to β-CATENIN protein as compared to WT1-WT (Fig. 3*D*). Two other variants, which affect ZF4 (WT1p.Arg495* and WT1p.Lys491Glu) also show significantly increased binding to β-CATENIN protein as compared to the WT1-WT protein. The variant in ZF3 (p.Gln437Lys), which is not associated with DSD in XX individuals, showed a low level of interaction with the β-CATENIN protein that is comparable to the WT1-WT (*SI Appendix*, Fig. S6), suggesting that the stronger interaction of WT1 proteins with β-CATENIN is a unique property of ZF4 mutants.

### A Mouse Model of WT1p.Arg495Gly Shows Masculinization of XX Embryonic Gonads.

**Fig. 4.**
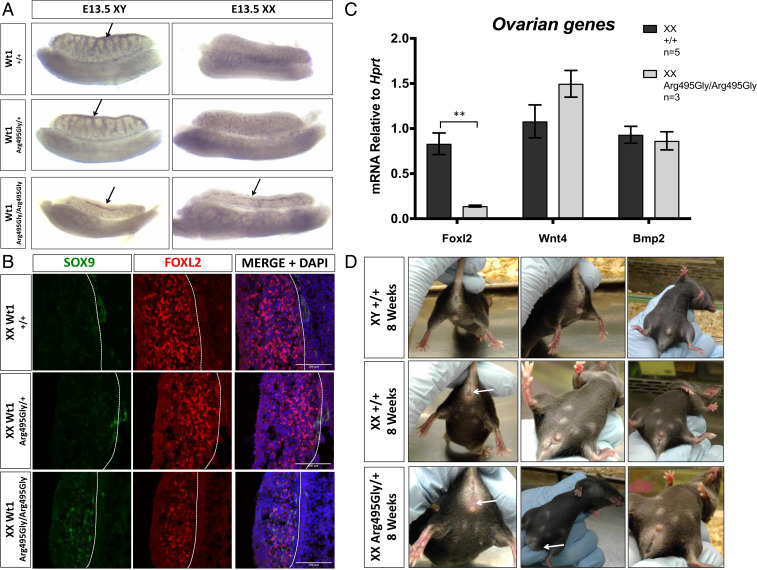
Phenotype of the *Wt1*^*Arg495Gly*^ mutant mouse model. (*A*) Bright-field images of WT (*Wt1*^*+/+*^), heterozygous (*Wt1*^*Arg495Gly/+*^), and homozygous (*Wt1*^*Arg495Gly/Arg495Gly*^) XY and XX gonads at E13.5. Black arrow indicates the presence of the coelomic vessel, which is normally only present in the testis. (*B*) Immunostaining of E13.5 gonads from XX WT, heterozygous, and homozygous mice. Gonads were stained for Sertoli-marker SOX9 (green), Granulosa-marker FOXL2 (red), and DAPI (blue). Homozygous XX gonads appear with less FOXL2-expressing cells and present SOX9-positive cells. (Scale bars, 100 μm.) (*C*) RT-qPCR of genes involved in female sex determination (*Foxl2*, *Wnt4*, and *Bmp2*) in XX WT and *Wt1*^*Arg495Gly/Arg495Gly*^ gonads at E13.5. Data are presented as mean 2^-ΔΔCt^ values, normalized to the housekeeping gene *Hprt*. Sample size represents the number of individuals and is indicated on the side. Error bars show SEM of 2^-ΔΔCt^ values. *P* value is presented above the relevant bars (unpaired, two-tailed *t* test on 2^-ΔΔCt^ values, *P ≤ 0.05, **P ≤ 0.01). Dark gray bars, WT XX gonads; light gray bars, *Wt1*^*Arg495Gly/Arg495Gly*^ homozygous XX gonads. (*D*) Images of the external genital anatomy of WT XY, WT XX, and heterozygous *Wt1*^*Arg495Gly/+*^ XX mice at 6 wk of age. These heterozygous XX mice present with no vaginal opening (indicated by white arrow on *Left*); a bulge between the anus and genitalia that usually appearing only in males (indicated by white arrow at *Center*); and clear presence of nipples. This was the result of vaginal atresia and hydrometrocolpos.

In order to better understand the role of ZF4 pathogenic variants to induce testis formation in an XX chromosomal context, we introduced a nonsynonymous change (*SI Appendix*, Fig. S7) into the endogenous mouse *Wt1* gene to create WT1p.Arg495Gly. The protein sequence of ZF4 is highly conserved between human and mouse including the arginine residue located at position 495 (*SI Appendix*, Fig. S2). We used CRISPR/CAS9 genome editing with a single-strand oligo (HDR ssOligo) to obtain homology-dependent repair and fully mimic the variant found in Patient 1 (*SI Appendix*, Fig. S7 *A*–*C*). As in the case of null mutations of *Wt1* (26), the XY homozygous *Wt1*^*Arg495Gly/Arg495Gly*^ gonads are thin and elongated and do not form testis cords (Fig. 4 *A*, *Left* and *SI Appendix*, Fig. S8 *A*, *Left*). XX homozygous *Wt1*^*Arg495Gly/Arg495Gly*^ gonads are also very thin, elongated, and partly masculinized with the appearance of a clear coelomic vessel (Fig. 4 *A*, *Right* and *SI Appendix*, Fig. S8 *A*, *Right*), which is normally found only in testes or in the masculinized gonads of XX mice with deletion of key ovarian genes, notably *Wnt4* or *Rspo1* (27, 28). Immunostaining of homozygous *Wt1*^*Arg495Gly/Arg495Gly*^ XX gonads with the granulosa cell marker FOXL2 and the Sertoli cell marker SOX9 confirmed a thinner gonad with fewer FOXL2 expressing cells when compared to WT and heterozygous gonads, as well as appearance of SOX9-positive cells (Fig. 4*B*). Staining with antibody against WT1 concurs with our in vitro observation, where the mutation does not prevent protein synthesis and localization within the nucleus (Fig. 2*E* and *SI Appendix*, Fig. S8*B*). Staining with the germ cell marker TRA98 confirmed the presence of germ cells in the gonads (*SI Appendix*, Fig. S8*B*).

To further characterize the partial masculinization observed in XX gonads, we performed qRT-PCR on XX gonads isolated from embryonic day (E) 13.5 embryos. Remarkably, homozygous *Wt1*^*Arg495Gly/Arg495Gly*^ gonads show more than an 80% reduction in the expression level of *Foxl2* (*P* < 0.005), a gene essential for maintaining ovarian identity (2). There was no change detected in the expression of other ovarian markers such as *Wnt4* or *Bmp2* (Fig. 4*C*). Expression levels of several testis markers (*Wt1*, *Sf1*, *Sox9*, *Dmrt1*, and *Fgf9*) show marginal increases, but this was not always statistically significant (*SI Appendix*, Fig. S8*C*). The XY homozygous *Wt1*^*Arg495Gly/Arg495Gly*^ gonads were also characterized by immunohistochemistry (*SI Appendix*, Fig. S9) and confirmed the absence of testis cords, with very few scattered SOX9-expressing cells and numerous FOXL2-positive cells. As in the XX gonads, the WT1 protein is strongly expressed and located within the nucleus.

The majority of heterozygous XX *Wt1*^*Arg495Gly/+*^ females appeared normal and were fully fertile, but we observed three females with an apparently masculinized phenotype (Fig. 4*D*). These animals were first identified at ∼6 wk, just when they reached sexual maturity. They presented with no vaginal opening, an enlarged bulge between the anus and genitalia similar to males, while still having nipples (Fig. 4*D*). However, gonadal dissection of these XX mice indicated the presence of relatively normal looking ovaries, while having an extremely enlarged uterus (*SI Appendix*, Fig. S8*D*). The vastly enlarged uterus phenotype and appearance of the bulge is probably a result of the lack of vaginal opening (vaginal atresia) and accumulation of vaginal secretions (hydrometrocolpos) and not necessarily the masculinization of the external genitalia.

Given the importance of *Wt1* in kidney development, we observed that age-matched kidneys in the homozygous *Wt1*^*Arg495Gly/Arg495Gly*^ mice (both XX and XY) are significantly smaller as compared to the WT and heterozygous animals. The cortex is architecturally homogeneous with no clear boundary between the outer cortex and juxtamedullary cortex. There is a significant lack of developed renal corpuscles, and glomerular spaces are not observed (details in *SI Appendix* and *SI Appendix*, Fig. S10).

Taken together, the partial sex reversal observed in the embryonic gonads of XX mice carrying the homozygous *Wt1*^*Arg495Gly/Arg495Gly*^ allele strongly supports our hypothesis that variants disrupting the ZF4 of WT1 cause gonadal masculinization in an XX chromosomal context in both humans and mice.

## Discussion

In a series of 78 children presenting with *SRY*-negative 46,XX TDSD/OTDSD, we identified 7 (9%) children with recurrent missense and frameshift pathogenic variants in the ZF4 domain of the *WT1* gene that result in a functional WT1 protein where this domain is either disrupted or absent. Where tested, the variants were de novo and all are absent from public single nucleotide polymorphism (SNP) databases and in-house fertile control cohorts suggesting the variants have complete penetrance. None of the patients had a Wilms’ tumor, nor renal anomalies, with the exception of Patient 7 where ultrasound examination showed small, cystic kidneys with suboptimal corticomedullary differentiation (*SI Appendix*, Fig. S1). However, focal segmental glomerulosclerosis, diffuse mesangial sclerosis, Wilms’ tumors, or gonadoblastoma may potentially develop among this cohort. In one family, a daughter presented with 46,XX ovotesticular DSD (Patient 5a) while her 46,XY brother, carrying the p.Arg495Gln variant, presented with characteristics of Meacham syndrome: partial gonadal dysgenesis and diaphragmatic hernia. This shows that the p.Arg495Gln variant is associated with DSD in both sexes.

WT1 encodes a complex transcription factor, with at least 36 potential isoforms, that contains four zinc-finger motifs at the C terminus (29). The variants identified in this study are all predicted to affect the ZF4 of WT1 protein (Fig. 2*A*). These are either missense variants of highly conserved amino acids (*SI Appendix*, Fig. S2) or frameshift variants that are predicted to result in the absence of ZF4. Previous studies have detected the expression of *Wt1* RNA in ovaries isolated from mice 17 d postpartum, where it is expressed at high levels in somatic cells of peripherally located follicles and in the epithelial cell layer of the ovary (30). In the human GW10 embryo, the strongest expression of the WT1 protein was observed in the epithelial cell layer surrounding the ovary, specifically in areas proximal to the paramesonephric duct (31). Here, we found that WT1 is widely expressed in both a FOXL2-negative stromal cell population and in FOXL2-positive granulosa cells in normal developing human XX ovaries (GW9+5, Fig. 2 *B*–*D*). The observed WT1 expression profile is strikingly similar to the expression profiles of the nuclear receptors NR2F2 and NR5A1 proteins in the developing ovary. Both of these factors, when mutated, cause 46,XX TDSD/OTDSD (16, 17). We can speculate that all these factors may function in the same developmental regulatory network. Although our**in vitro data does not show a direct interaction between WT1 and the nuclear factors, we cannot exclude that they may act synergistically or sequentially on common targets at the time of embryonic gonad determination or form part of the same regulatory pathway(s).

Using WT1p.Arg495Gly as a proof of concept, we investigated the functional consequences of the ZF4 variants and the disease mechanism using in vitro studies, in situ assays, as well as a mouse model. The WT1p.Arg495Gly protein localized to the nucleus and maintained its DNA binding activity; however, it showed a context-dependent alteration in its transactivation potential and protein-binding ability. Vertebrate sex determination is regarded as a bistable double repressive system where mutual antagonism exist between protestis and pro-ovary signaling pathways. Thus, 46,XX TDSD/OTDSD can be viewed as an imbalance between these competing regulatory networks leading to increased expression of protestis genes in the XX embryonic gonad, over a threshold level required to establish testis formation. This concept is recapitulated in the in vitro assays in which the mutant WT1 increased the expression of protestis factors and showed a reduced ability to activate the pro-ovary gene *FOXL2*. The aberrant regulation seen with the in vitro reporter assays and the activation of protestis transcripts in the granulosa cell line are seen only when the ZF4 variants are present with the WT WT1, and not by either the WT or mutant protein alone. Therefore, the formation of testis in the XX gonad may be due to a dominant negative effect of the ZF4 variants on the biological activity of the WT WT1. This activity was not observed with a variant in ZF3 that is associated with renal disease, but not XX DSD, in a pair of female monozygotic twins (22). Accordingly, ZF4 mutants may be regarded as gain-of-function rather than LOF, and these observations may explain why variants in other regions of the protein do not result in formation of testicular tissue in 46,XX individuals.

In a previous study, *Wt1* was ablated at ∼10.5 d.p.c. (days post coitum), which resulted in a differentiation block of the supporting cell lineage, together with most somatic cells acquiring markers of steroidogenesis in both XX and XY gonads (26). The number of FOXL2-positive cells was also dramatically reduced in *Wt1*-deficient XX gonads, and very few SOX9-positive cells were observed. There was no testicular cord formation in *Wt1*-deficient XX gonads (26). Here, the embryonic gonads of homozygous XX *Wt1*^*Arg495Gly/Arg495Gly*^ mutant mice showed distinct signs of masculinization, including the formation of a testis-specific coelomic vessel, the appearance of numerous SOX9-positive cells, together with an extensive reduction in FOXL2-positive cells. The gonadal phenotype observed in the homozygous mice was not seen in the heterozygous animals. This is not surprising since in many instances, heterozygous gene variants associated with DSD in human show sex reversal in mice only when the gene is either completely absent or overexpressed involving multiple copies (32). This may reflect differences in gene dosage thresholds in sex-determination networks between the mouse and human. Hence, the mouse model may not conform with a “gain-of-function” explanation for the human phenotypes as suggested by experiments on human cell lines in vitro. Nevertheless, it does provide an in vivo system that supports the importance of ZF4 of the WT1 protein for normal sex determination of XX individuals. In addition, whereas XX *Wt1*^*Arg495Gly/*^ ^*Arg495Gly*^ homozygous mice show a marked decrease in *Foxl2* mRNA levels, the KGN-1 cells cotransfected with the mutant and WT variants did not show down-regulation of pro-ovary genes, but rather the up-regulation of protestis genes. This difference may stem from the gene dosage thresholds mentioned above and/or reflect the static state of the cells in culture versus a dynamic developing system in vivo. That being said, we cannot exclude that the mechanism of action of the WT1p.Arg495Gly variant differs between human and mouse but, in both, still leading to development of XX ovotestes or testes.

β-CATENIN plays an essential role during human and mouse ovarian development (33, 34). Maintenance of β-CATENIN signaling by RSPO/WNT is required not only to support or promote the ovarian pathway, but also to repress SOX9 expression and the male pathway (28, 33). In vitro, we observed that ZF4 variants not only increase the activation of a relevant *Sox9* enhancer, but also shows binding to the β-CATENIN protein, whereas the WT protein does not. Remarkably, a variant in ZF3 (p.Gln437Lys), which is associated with renal disease but not DSD in XX individuals (22), showed a low level of interaction with the β-CATENIN protein that is comparable to the WT1-WT. By altering the ability of WT1 to interact and apparently interfere with the essential pro-ovary and antitestis factor β-CATENIN, the ZF4 variants could result in up-regulation of SOX9 expression. Based on these data, we propose a mechanism to explain testis formation in XX individuals carrying WT1 ZF4 variants. The interaction between the mutant WT1 and β-CATENIN proteins results in sequestration of β-CATENIN that negatively impacts on the activation of the pro-ovary pathways and/or results in an absence of SOX9 repression. Consequently, a protestis signaling cascade is activated in an XX chromosomal context. However, the biology of WT1 is highly complex with multiple isoforms showing distinct subnuclear localizations, and diverse regulatory properties at both the transcriptional and posttranscriptional levels (29, 35–38). It cannot be excluded that the mutant WT1 may induce testis formation in XX gonad by other mechanisms that include RNA and chromatin modifications.

In conclusion, while reflecting a complex early role for WT1 in gonadal development in both sexes, but in contrast with other variants of WT1, we propose that the variants we discovered in the ZF4 domain of WT1 may result in either direct or indirect repression of the female pathway and activation of the male pathway during embryonic development of the XX gonad. These mutations, therefore, are a relatively frequent cause of 46,XX SRY-negative (O)TDSD.

### Data Availability Statement.

All data discussed in the paper are available in *SI Appendix*.

## Supplementary Material

Supplementary File
