# Supplementary material for: Testis formation in XX individuals resulting from novel pathogenic variants in Wilms’ tumor 1 (WT1) gene
Source: Proc Natl Acad Sci U S A. 2020 Jun 3;117(24):13680–8. doi: 10.1073/pnas.1921676117 (PMC7306989; doi:10.1073/pnas.1921676117)
Supplement: Supplementary File [file pnas.1921676117.sapp.pdf]

## SUPPLEMENTARY APPENDIX

## Material and method

### Extended Clinical data.

Patient ancestry was determined by self-reporting, based on responses to a personal questionnaire, which asked questions pertaining to the birthplace, languages and self-reported ethnicity of the participants, their parents and grandparents. Genes known to be involved in 46,XX DSD were screened for mutations in the XX DSD cohort and high resolution aCGH was performed on all cases that indicated normal ploidy for each patient.

Patient 1 is a 14 years-old child of Egyptian Origin, of consanguineous parents with unremarkable family history and assigned male sex-of-rearing. He presented with ambiguous genitalia. General examination showed no dysmorphic features, normal weight and height with small sized head circumference (-4.5SD). On genital examination gonads were not palpable. He had a penis-like phallus (length 9cm), single urethral opening and labioscrotal folds. Pubertal Tanner stage 2, pubic hair stage 4 and axillary hair stage 3. Karyotype was 46,XX and fluorescence in situ hybridization (FISH) with *SRY*-specific probes excluded an *SRY* translocation. Biochemical testing showed serum testosterone level of 0.9 ng/ml (reference value female 0.1:1ng/ml), estradiol 13 pg/ml (reference 20:60pg/ml), FSH 105 mIU/ml (reference 20 mIU/ml) and LH 81 mIU/ml (reference 24 mIU/ml). Pelvic ultrasound showed a prepubertal uterus behind urinary bladder. Gonads could not be visualized. He had laparoscopic excision of bilateral pelvic gonads and uterus and pathology revealed a left fibrotic nodule (1.5 x 1.5 cm), a right fibrotic nodule (2 x 2cm) and a small uterus (4 x2 cm). Histopathology indicated on both right and left sections, fibrosed and hyalinized tubules as well as tubules lined by Sertoli cells with no evidence of germ cells. There was marked interstitial cellular hyperplasia. Uterus sections showing muscular uterine wall lined by inactive endometrium and no evidence of ovarian tissue or malignancy (Table 1; Figure 1A).

Patient 2 is a 2.5-years-old child, the second of healthy nonconsanguineous parents with unremarkable family history and assigned female sex of rearing. Ambiguous genitalia

was observed at birth and she was referred with diagnosis of congenital adrenal hyperplasia (CAH) due to 21-hydroxylase deficiency because of increased serum 17Hydroxyprogesterone levels. However, molecular study of *CYP21A2* gene was negative. Genital examination showed 3 cm length phallus, single perineal orifice, scrotal folds fused in midline and non-palpable gonads. External masculinization score (EMS): 7/12. Karyotype was 46,XX and FISH excluded an *SRY* translocation. A human chorionic gonadotropin (hCG) stimulation test was performed at age of 3.5 years, resulting in a serum testosterone level of 2.37 ng/ml and anti-Müllerian hormone (AMH) of (34 pmol/l), both in female reference range. Müllerian structures were detected on ultrasound. Laparoscopy and bilateral gonadal biopsy showed the presence of testicular tissue. Histology confirmed the presence of testicular parenchyma with signs of dysgenesis and scarce germ cells. The patient was lost in follow-up (Table 1).

Patient 3 is the first child of nonconsanguineous healthy parents, born full-term with adequate weight birth. Familial history is unremarkable. At birth genital ambiguity was noticed. Cytogenetic studies showed a 46,XX *SRY*-negative karyotype. The baby was assigned the female sex. Genital examination at 3 months of age showed 2.2 cm length phallus with chordee, complete labio-scrotal fusion, single and small peno-scrotal orifice and nonpalpable gonads. EMS: 4/12. Testosterone after hCG stimulation test was 3.79 ng/ml; AMH was between the male and female reference values (164.2 pmol/l) and GnRH stimulation test showed a predominant LH response (29.4 MIU/ml). Müllerian structures were detected on ultrasound. Laparoscopy revealed intra-abdominal gonads and two hemi Müller. Histology of bilateral gonadal biopsy showed dysgenetic testicular parenchyma with scarce germ cells. The baby was reassigned the male sex at 3 months of age. Hypospadias repair and orchidopexy were performed between 1-3 years of age (Table 1; Figure 1B).

Patient 4 is the first child of healthy nonconsanguineous parents. Infertility was reported in some relatives of the baby´s father. At birth ambiguous genitalia was observed. On genital exam the patient presented a 3 cm length phallus with well-developed corporal tissue, pigmented labioscrotal folds fused in the midline, a single phallo scrotal orifice and nonpalpable gonads. EMS: 7/12. Karyotype was 46,XX and *SRY*-negative. At 12 days of life, testosterone after hCG stimulation test was 4.91 ng/ml; AMH was low for the male reference range (82.1 pmol/l) and GnRH stimulation test showed a

predominant LH response (20.2 MIU/ml). Pelvic ultrasound revealed an apparently normal uterus and both gonads with follicles. Laparoscopy and bilateral biopsy were performed. Histological analysis showed that both gonads were ovotestes with gonadoblastoma and dysgerminoma and gonadectomy was performed. The patient was assigned the female sex (Table 1; Figure 1C).

Patient 5a was born at 38 weeks of gestation after an uneventful pregnancy. Baby was a first child of healthy nonconsanguineous Hungarian parents. Atypical genitalia was noticed at birth, with 3 cm length and 1.5 cm wide phallus like clitoris, single penoscrotal orifice, complete labioscrotal fusion and non-palpable gonads (Prader IV, EMS 7/10). Congenital adrenal hyperplasia was excluded and the cytogenetic testing revealed a 46,XX, *SRY*-negative karyotype. The gender of rearing was female. At three months of age explorative laparotomy identified a uterus and two macroscopically undifferentiated gonads. At the age of six month, she underwent feminization genioplasty, her androgen levels were still significantly elevated with testosterone 152 ng/dl (2-15 ng/dl) and androstenedione 81 ng/dl (5-40 ng/dl). At the age of four years the basal testosterone level was low (<10 ng/dl) and in the hCG stimulation test it reached 42 ng/dl. At age of 12 years she underwent a bilateral gonadectomy and histological examination revealed ovotestis in both gonads without germ cells or sign of malignancy. A diagnosis of 46,XX ovotesticular DSD was indicated (Table 1; Figure 1D).

Patient 5b is the brother of Patient 5a, was born at 38 weeks of gestation. His birth weight was 3,200 g and birth length was 57 cm. At birth he presented with male external genitalia, but testes were not palpable in the scrotum nor in the inguinal canal. After birth he underwent surgery for a diaphragmatic hernia. His karyotype was 46,XY. At age 1.2 years his basal FSH was 0.7 IU/L and testosterone <0.1 nmol/L, but after the hCG stimulation the testosterone level increased properly (11.1 nmol/L). At 1.8 years of age laparoscopy identified a rudimentary testis on the right side. Orchidectomy was performed on the left side and histological examination of this gonad did not find any testicular tissue, but pieces of funiculus spermaticus and epididymis tissue. At age of 9 years his hormone levels were in prepubertal range (FSH: 0.6 IU/L, LH <0.11 IU/L, T < 0.43 nmol/L, E2 < 92 pmol/L). The combination of diaphragmatic hernia with 46,XY DSD indicated a diagnosis of Meacham syndrome (Table 1; Figure 1E).

Patient 6 was first seen in the clinic as a 32-years old infertile man. There is no history of infertility in his family. General appearance and hair distribution were male. Physical examination showed testis size of 8 cc and both gonads were palpable. The patient had bilateral hernia. He had right epididymis orchitis followed by orchiopexy surgery in childhood. Karyotype was 46,XX and *SRY*-negative. Serological test showed increased gonadotrophins (FSH (25.80 mIU/mL [1.5–12.4]) and LH (27.93 mIU/ml [1–10])) and testosterone (1.32 ng/mL [2–8]). Semen analysis showed azoospermia (Table 1).

Patient 7 is an 8 years-old Caucasian child, born at term to a 23 year old G2P1 mother. Both parents were healthy with unremarkable family history and no evidence of consanguinity. The congenital diaphragmatic hernia was identified prenatally. At birth child presented with atypical genitalia and congenital diaphragmatic hernia that prompted chromosome analysis, endocrine evaluation, and molecular testing for the *WT1* gene to rule out Denys-Drash syndrome. Physical examination showed enlargement of the phallus, perineal hypospadias, single perineal opening, and complete fusion of the labia. No gonads were palpable in the labioscrotal folds or inguinal regions. In the newborn period, 17-OHP was 71 ng/dl. Imaging studies showed a urogenital sinus with low confluence and bicornate uterus. Karyotype showed normal female 46,XX chromosome complement, and array CGH analysis was negative for the Y chromosome DNA sequences and copy number variants (Table 1). Child was assigned female sex of rearing and underwent feminizing genitoplasty. At 8 years, patient's weight was 17.6 kg (-2.94SDS) and height was 115.3 cm (-2.4SDS). Most recent abdominal and pelvic ultrasounds showed small, cystic kidneys with suboptimal corticomedullary differentiation, bicornuate uterus, and normal appearing gonads presumed to be ovaries. Testosterone and androstenedione were undetectable. Thyroid function studies were normal. FSH was 5.25 mIU/ml and LH was 0.05 mIU/ml. Her IGF-1 was 67 ng/ml which is just at 2SD below mean for age. No gonadal biopsies were performed (Table 1).

**Whole Exome sequencing.**

78 patients presenting with 46,XX (O)TDSD were sequenced using the whole exome approach. Exon enrichment was performed with Agilent SureSelect Human All Exon V4 or V5. Paired-end sequencing was performed on the Illumina HiSeq2000 or

HiSeq2500 platform with TruSeq v3 chemistry. Read files (fastq) were generated from the sequencing platform via the manufacturer's proprietary software. Reads were mapped with the Burrows-Wheeler Aligner, and local realignment of the mapped reads around potential insertion/deletion (indel) sites was carried out with GATK version 1.6. Duplicate reads were marked with Picard version 1.62 (http://broadinstitute.github.io/picard/). Additional BAM file manipulations were performed with Samtools (0.1.18). SNP and indel variants were called with the GATK Unified Genotyper for each sample. SNP novelty was determined against dbSNP138. Novel variants were analyzed by a range of web-based bioinformatics tools on EnsEMBL SNP Effect Predictor (http://www.ensembl.org/homosapiens/userdata/uploadvariations). All variants were screened manually against the Human Gene Mutation Database Professional Biobase (http://www.biobase-international.com/product/hgmd/). *In silico* analysis was performed to determine the potential pathogenicity of the variants. Potentially pathogenic mutations were verified with classic Sanger sequencing.

**Homology alignment and modeling of the 2-D structure of WT1.**

All protein sequences were obtained from the Ensembl database (http://www.ensembl.org),

Human- ENST00000332351.7
Macaque- ENSMMUT00000013806.3
Mouse- ENSMUST00000143043.7
Rat- ENSRNOT00000067940.3
Rabbit- ENSOCUT00000005884.3
Dog- ENSCAFT00000011914.3
Horse- ENSECAT00000030647.1
Goat- ENSCHIT00000018366.1
Sheep- ENSOART00000017787.1
Chicken- ENSGALT00000019788.6
Zebrafish - ENSDART00000139962.2
Xenopus- ENSXETT00000017924.3

Sequence alignment was performed using the web based multiple sequence alignment software Clustal-W (www.ebi.ac.uk/clustalw/).

In silico analysis predicts a disruptive effect of sequence variants in the ZF4 of WT1 on DNA binding. An in-silico 3D model was created using the X-ray crystallographic structure of human Zinc fingers 2-4 wild-type WT1-DNA complex (40). Models of the zinc finger domains of WT1 KTS+ (PDBID 6BLW) or KTS- (PDBID 6B0R) with DNA were generated with the Pymol Molecular Graphics System (Schrödinger LLC). Figures of the Superposition of ZF4 of the KTS- structure on the partial ZF4 structure of the KTS+ structure was performed with Lsqkab (40, 41). In an effort to minimize overinterpretation of the structural alignment, no refinement was performed. Predictive structures were analysed for the p.Lys491Gln, p.Arg495Gly and p.Arg495Gln proteins.

**Plasmid construction.**

Vector containing full-length WT1(+KTS) isoform in a pSG5 vector was kindly provided by Dr Maëlle Pannetier and Dr Eric Pailhoux. The pCMX-NR5A1 vector with human NR5A1, the pCMV6-FOXL2, the pIRES-hrGFPII-Gata4, pCS2+Fog2, pCDNA-SOX9-Flag and Tesco reporters have been previously described (42-44). To assay the ability of WT1 proteins to modulate the canonical WNT pathway, we used the TOPFlash-TCF reporter plasmid, as previously described (16). Vector containing a 1-kb fragment of the caprine FOXL2 promoter from - 842 to +168 was cloned upstream of the luciferase gene in a pGL3basic vector (Promega) and have been previously described (45) and kindly provided by Dr Maëlle Pannetier and Dr Eric Pailhoux.

**Site-Directed Mutagenesis**.

WT1 expression vectors containing the p.Arg495Gly mutation was generated by site-directed mutagenesis (QuikChange, Stratagene) with the use of wild-type (WT) mouse *Wt1* cDNA in a pSG5 expression vector as a template. The entire coding sequence of all mutant plasmids was confirmed by direct sequencing prior to functional studies.

**Cell lines.**

HEK293-T, human embryonic kidney (HEK) 293-T cells as well as KGN, human granulosa-like tumor cell line (Nishi et al., 2001) were used in this study. For all

transfections, HEK293-T and KGN cells were transfected using FuGENE® HD Transfection Reagent (Promega) and GeneJuice® Transfection Reagent (Merck Millipore) respectively according to manufacturers' recommendations,

Transient gene expression assays for the assessment of WT1 function were performed in 96well plates for luciferase transactivation assays (Eppendorf or TPP) with the use of a DualLuciferase reporter assay system (Promega) with pRLSV40 Renilla luciferase (Promega) expression as a marker of transfection efficiency, in 6-well plates for western blot and coimmunoprecipitation assays (Eppendorf or TPP) and in Chamber slides (Nunc) for immunofluorescence and Duolink Proximity Ligation assays.

**Luciferase transactivation assays.**

pSG5-WT or mutant WT1 expression vectors (10 ng/well) were co-transfected into HEK293-T cells with reporters containing WT1 responsive minimal promoter (caprine FOXL2) or enhancers (murine Tesco, TOPFlash-TCF reporter) (10 ng/well) and with pCMX-NR5A1, the pCMV6-FOXL2, the pIRES-hrGFPII-Gata4, pCS2+Fog2, pCDNASOX9-Flag expressing vectors (10ng/well). Cells were lysed 48 hr later, and luciferase assays were performed with the use of a Glomax microplate reader MultiDetection system (Promega). All data were standardized for Renilla activity. Results are shown as the mean ± SEM of at least three independent experiments, each performed at least in quadriplicate.

**Transfections in KGN-1 cells line.**

Using GeneJuice® Transfection Reagent, 500ng or 1ug of plasmids were transfected in KGN cells (WT1-wild-type, WT1p.Arg495Gly and empty vectors). After two-days of transfection, cells were collected in TRIzol™ Reagent (Invitrogen™) and stored at -80°C prior to analysis. RNA extraction was performed according to the manufacturer recommendations. Both quality and quantity were assessed through the Agilent 2100 Bioanalyzer System (Agilent Technologies).

**RNA preparation.**

Total RNA was extracted from transfected KGN-1 cells and E13.5 gonad pairs using the RNeasy Plus Micro kit (Qiagen). RNA yield was quantified with a NanoDrop spectrophotometer (NanoDrop Technologies), and 200 ng RNA was used to synthesize cDNA with the SuperScript VILO™ cDNA Synthesis Kit (Invitrogen) or Quantitect Reverse Transcription Kit (205311,. QIAGEN) as per manufacturer's recommendations

**Quantitative Real-Time Polymerase chain reaction (qRT-PCR) for human Granulosa tumour cell line (KGN-1).**

cDNA was synthesised using 500ng of RNA. cDNA was diluted 1/25 prior the TaqMan real-time PCR. TaqMan probes; RPL19: Hs02338565_gH; SOX9: Hs01001343_g1; DMRT1: Hs00232766_m1; NR5A1: Hs00610436_m1; Human WT1: Hs01103751_m1; Mouse Wt1: Mm01337048_m1, FOXL2: Hs00846401_s1; β-catenin: Hs00355049_m1 FST: Hs01121165_g1. qPCR was performed using TaqMan Universal Master Mix II, with UNG (4440038, Applied Biosystems) on a StepONEPlus qPCR machine (Applied Biosystems). Relative mRNA levels were determined by calculating 2-ΔΔCt values relative to the 18S rRNA normalizer gene (RPL19). Relative gene expression is presented as the mean 2-ΔΔCt values (error bars are SEM of the 2-ΔΔCt).

**Collection of human fetal gonads and Immunofluorescence.**

Human fetal ovaries were isolated from material available following elective termination of pregnancy during the first trimester at the Department of Gynaecology at Copenhagen University Hospital (Rigshospitalet) and Hvidovre Hospital, Denmark. Fetal age determined by scanning crownrump length and by evaluation of foot length (46). Sex of foetal samples was determined by PCR for *SRY* as previously reported (47).

**Immunofluorescence on human fetal gonads**.

Tissue was fixed in formalin immediately after dissection and dehydrated, paraffin embedded and sectioned (4 μm) using standard procedures. Immunofluorescence was performed as previously described (48). Primary antibodies used was WT1 (Abcam,

Ab89901, diluted 1:1000) and FOXL2 (a kind gift from Dr. Dagmar Wilhelm, diluted 1:100). Negative controls were included and processed with the primary antibody replaced by the dilution buffer alone. None of the negative control slides showed staining. Fluorescent images were captured using an Olympus BX61 microscope (Olympus) with the Cell Sens Dimension software version 1.16.

**Cellular Localization.**

Cellular localization of both wild-type WT1 and mutants were assayed by transfecting the different plasmids into HEK293-T cells with the use of FuGENE 6 (Roche) in chamber slides (Nunc). Cells were fixed in 4% paraformaldehyde in PBS (pH 7.4) for 15 min at room temperature, 48 hrs post transfection, washed twice with ice cold 1xPBS, permeabilized in 1xPBS containing 0.5% Triton X-100, and then incubated with 5% BSA in PBS for 30 min to block non-specific binding of the antibodies. Cells were incubated with the primary antibody (diluted in 3% BSA in PBS) in a humid chamber overnight at +4°C. The following dilutions of primary antibodies were used: anti-WT1 and anti-β-catenin (H00001499-M06, Abnova), 1:100. After 16 h incubation, cells were washed three times with 1xPBS for 5 min each. This was followed by incubation with the secondary antibody in 3% BSA for 1h at room temperature in the dark Goat anti-Rabbit IgG (H+L) Secondary Antibody, Alexa Fluor® 488 conjugate, #A11034, and Goat anti-Rabbit IgG (H+L) Secondary Antibody, Alexa Fluor® 594 conjugate #A11037, Goat anti-mouse IgG (H+L) Secondary Antibody, Alexa Fluor® 488 conjugate, #A11029, Donkey anti-mouse IgG (H+L) Secondary Antibody, Alexa Fluor® 594 conjugate, #A21203, Life Technologies, Donkey anti-goat IgG (H+L) secondary antibody, Alexa Fluor® 488 conjugate, #ab150129, Abcam, diluted to 1:1000), cells were washed three times with 1xPBS for 5 min each in the dark. Slides were then mounted using ProLong® Gold Antifade Mountant with DAPI. Images were obtained with a Leica Microsystems DMI4000B microscope at 40x or 63x magnifications.

**Gel shift assay with biotinylated probe and in vitro translated WT1 proteins.**

Wild-type WT1 and WT1p.Arg495Gly were in vitro translated using the TNT system (Promega) as per the manufacturer's instructions. Protein binding to target DNA probes

was assessed by the electrophoretic mobility shift assay (EMSA). The oligos used for the EMSA were Biot_WT1_BS_for (5' TEG-biotin): TCCTCTATCCCCTCCCCTCTCCTCTCCTTT and Biot_WT1_BS_rev (5'-TEG biotin): AAAGGAGAGGAGAGGGGAGGGGATAGAGGA. The 5' TEG biotin oligos were hybridized (50 µg per oligos) in 10mM Tris ph7.5, 1mM EDTA, 50 mM NaCl, by incubated in boiling water bath for 5 minutes and allowed to slowly cool to room temperature. The hybridized probe was purified on a 6% nondenaturing acrylamide gel and eluted overnight in 0.5 M ammonium acetate–10mM Magnesium acetate-5 mM EDTA–0.1% SDS buffer at 50°C and concentrated by ethanol precipitation. Probe was quantified using Qubit fluoroimager (Invitrogen). For the binding reaction, 2 µl of programmed TNT lysate was mixed with 50 fmol of purified probe and 2 µg of poly(dI-dC) in 20-µl final volume of binding buffer (20 mM HEPES [pH 7.9], 5% glycerol, 0.1 M KCl, 0.1mM ZnSO4, 0.5 mM DTT). The DNA-protein complexes were resolved by electrophoresis on a 10% polyacrylamide gel in 0.5X Tris-borate-EDTA buffer at 4°C and visualized using the chemiluminescent nucleic acid detection module (PIERCE-thermoscientific ref: 89880) with no modification of the protocol.

**Duolink proximity ligation assay.**

Protein-protein interaction was detected by Duolink proximity ligation assay (PLA) Kit (Sigma-Aldrich: PLA Probe anti-rabbit plus; PLA Probe antimouse minus, PLA Probe anti-goat minus, Detection Kit DUO92014). The PLA probe antirabbit plus binds to the WT1 (sc-192, SantaCruz) antibody, whereas the PLA probe antimouse minus binds β-catenin antibody (β-catenin, H00001499-M06, Abnova) and the PLA probe anti-goat minus binds to the FOXL2 antibody (Ab5096, Abcam). If the distance between both proteins is <40 nm, a signal with DuoLink PLA is generated, thus indicating an interaction of both proteins. After pre-incubation with blocking agent for 1 h, transfected HEK293-T cells were incubated 2 hours at room temperature with the primary anti-GATA4 (1/100), anti- β-catenin (1/100), anti-SOX9 (1/100), anti-FOXL2 (1/100) antibodies then overnight at 4°C, with the primary anti-WT1 and anti-NR5A1 or anti-MYC antibodies (1/100). Duolink PLA probes detecting rabbit, mouse or goat antibodies were diluted in the blocking agent to a concentration of 1:5 and applied to the slides, followed by incubation for 1 hour in a humidity chamber at 37°C. Unbound

PLA probes were removed by washing. For ligation of the two Duolink PLA probes, Duolink ligation stock (dilution 1:5) was used. The cells were incubated in the ligation solution consisting of Duolink Ligation stock (1:5) and Duolink Ligase (1:40) for 30 min at 37°C. Detection of the amplified probe was done with the Duolink Detection Kit. Duolink Detection stock was diluted at 1:5 and applied for 100 min at 37°C. Slides were then mounted using ProLong® Gold Antifade Mountant with DAPI.

**Statistical analysis.**

Statistical analyses were carried out using GraphPad Prism 6 software (GraphPad Software). Quantitative data were subjected to a one-way ANOVA (or student ttest) followed by Bonferroni comparison.

**Design, preparation and microinjection of sgRNAs mRNA.**

Single guide RNA (sgRNAs) was designed (www.crispr.mit.edu) to target in proximity to the desired SNP, located in exon 10 of the mouse *Wt1* gene (MN_144783, chr 2), by CRISPR/Cas9 genome editing. sgRNA was prepared as described elsewhere (49). Briefly, the complementary pair of oligos (Table S1) was phosphorylated, annealed and cloned into pX459-V2 plasmid (Addgene #62988). A T7-sgRNA PCR product was amplified with a T7 promoter sequence introduced on the forward primer in conjunction with a universal reverse primer sgRNA-uni.R (Table S1). This product was used as the template for in vitro transcription (IVT) using the MEGAshortscript T7 IVT kit (Life Technologies). The sgRNA was purified using the MEGAclear kit (Life Technologies) and eluted in RNase-free water. Cas9 mRNA was obtained commercially (Tebu-Bio). Single-strand DNA oligo (ssOligo) (120 bp) was designed so that it contains the desired mutation of a G instead of a C, converting amino acid Arginine at position 495 into a Glycine. The oligo also contained 3 silent mutations that alter the DNA sequence of exon 10 but will not change the amino acid sequence of the WT1 protein (Figure S5). The silent mutations were inserted in order to prevent from the CAS9 protein from cutting the homology dependent repair (HDR) oligo that was inserted instead of the original Wt1 sequence. The ssOligo was synthesized by IDT. A mix of ssOligo sgRNA and CAS9 mRNA (100 ng/µl ssOligo, 50 ng/µl sgRNA, 100 ng/µl CAS9 mRNA) were injected into the cytoplasm of F1 (C57BL/6J x CBA) one-cell stage embryos as

described (49). Injected zygotes were surgically transferred on the same day into oviducts of pseudopregnant CD1 recipient females.

**Genotyping the genetically altered mice.**

Founders with the targeted insertion of the HDR allele were identified amongst live born mice using miSeq next generation sequencing of a PCR fragment surrounding the targeted region (all genotyping primers are listed in Table S1). Both founders and F1 mice were genotyped using miSeq. The following generations of mice were genotyped using either Sanger sequencing of a PCR fragment that contains the desired SNP/wild-type allele or using real time PCR with specific probes designed for the SNP/wild-type allele (Transnetyx, Cordova, TN). This was performed using genomic DNA extracted from tail tissue of embryos or ear punch tissue of adult animals. Chromosomal sex was determined by PCR genotyping with primers Sex-F and Sex-R (50) or using real time PCR with specific probe targeting the Y chromosome (Transnetyx, Cordova, TN). Two founders that contained one HDR-targeted allele and a second wild-type allele were used to transmit the HDR allele and a stable line was generated (Figure S5) Carrying the Arg495Gly WT1 mutation in two copies is embryonic lethal and embryos die just before birth and no adult homozygous mouse was even generated. Hence, homozygous embryos were generated by breeding two heterozygous mice.

**Next-generation sequencing to screen CRISPR deleted mice.**

Illumina MiSeq platform was used to sequence founder mice, because they are often mosaic, with several different alleles. The F1 mice were sequenced in the same way. The primers used for Sanger sequencing of the WT1 SNP PCR were also used for the miSeq PCR, however the adapter sequence was added to the 5’ and 3’ ends of the forward and reverse primers, respectively (Table S1, adaptor sequence in red), in order to enable further amplification with Illumina TruSeq indexing primers (Illumina). PCR was performed using Phusion High Fidelity PCR mix (New England Biolabs) according to the manufacturer’s protocol. Indexing was performed as per the Illumina protocol, and a clean-up step was done using AMPure XP beads (BeckmanCoulter). For sample quantification we used the dsDNA dye Quanti-fluor (Promega) and signals were recorded with Infinite M1000 Pro (Tecan) and analysed using proprietary Tecan

software. Samples were then normalised and pooled before submission to the Advanced Sequencing Facility (ASF) at the Francis Crick institute. Following quality control, involving library quantitation via Eco Real-Time PCR (Illumina) and sizing via 2100 Bioanalyzer (Agilent), the library was loaded onto the MiSeq at 2nM, using MiSeq Reagent Kit v2 (500 cycles) (Illumina) for 2 x 250bp sequencing. Fastq files were collapsed using FastX Toolkit (v0.0.13) and aligned to the reference genome (mm10) using the NCBI blastn.

**Timed mating and tissue preparation.**

Embryos and animals carrying *Wt1* SNPs were produced by crossing heterozygotes. Embryos were collected after timed matings at embryonic day E13.5, with noon of the day of plug designated as E0.5. For immunostaining, gonads from E13.5 embryos were harvested and fixed overnight in 4% paraformaldehyde in phosphate-buffered saline (PBS) at 4°C, washed three times with PBS at 4°C, then dehydrated and embedded in paraffin or OCT. The embedded samples were sectioned for immunofluorescence (OCT) or haematoxylin and eosin staining (paraffin). For RNA analysis of E13.5 embryos, paired gonad-mesonephros complexes (urogenital ridges) were dissected out, followed by sub-dissection of the gonad portion. A pair of gonads were snap frozen and stored in -80°C until further use for RNA isolation and gene expression analysis by qRT-PCR.

**Haematoxylin and eosin (H&E) and immunofluorescence staining.**

H&E histological staining was performed on 5μm-thick sagittal sections according to standard protocols. For immunofluorescence, staining was performed on 10 μm-thick sagittal cryostat sections, as described (Gonen et al., 2017) using the following primary antibodies: Rabbit polyclonal antiSOX9 IgG antibody (1:300, gift from Francis Poulat), Goat polyclonal anti-SOX9 IgG antibody (1:300, R&D Systems, AF3075), goat anti-FOXL2 (1:250, Novus, NB100-1277) and Rat antiTRA98 (1:200, Abcam AB-82527). Secondary antibodies were donkey anti-rabbit Alexa Fluor 488 (1:500, Invitrogen), donkey anti-goat Alexa Fluor 568 (1:500, Invitrogen). All immunofluorescence slides were also stained with 4',6-diamidino-2-phenylindole (DAPI, Molecular Probes), to visualize nuclear DNA.

**Quantitative Real-Time Polymerase chain reaction (qRT-PCR)**.

qRT-PCR reactions were performed in duplicate using SYBR Green PCR master mix (Invitrogen) and 150 nM each of forward and reverse primers, and analyzed on the Applied Biosystems 7500 Real-Time PCR System (Thermo Fischer Scientific). Primers are listed in Table S2. Relative mRNA levels were determined by calculating 2-ΔΔCt values relative to the normalizer gene Hprt. Relative gene expression is presented as the mean 2-ΔΔCt values (error bars are SEM of the 2-ΔΔCt) for multiple pairs of gonads from individual embryos (sample sizes as indicated on charts). Statistical analysis was performed using unpaired, two-tailed t-tests on the 2-ΔΔCt values.

## Supplementary Results

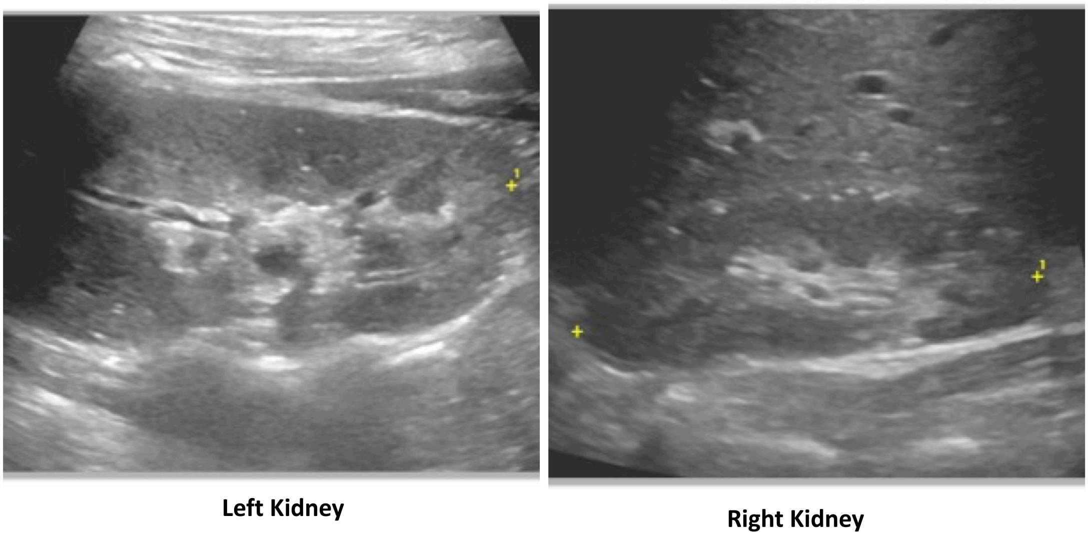


**Figure S1. Kidney Ultrasound for Patient 7.**
The kidneys of patient 7 are symmetrically small and show decreased corticomedullary differentiation. Punctate echogenic foci are noted throughout both kidneys. No abnormal septations or calcifications are noted. The normal renal size for her chronologic age is 8.9 ± 0.88 cm. No pelvocaliectasis or ureterectasis was noted. The right kidney measures 6.6 cm in length (yellow indicators). The left kidney measures 6.4 cm in length (yellow indicators) and contains several sub-centimetre simple renal cyts.

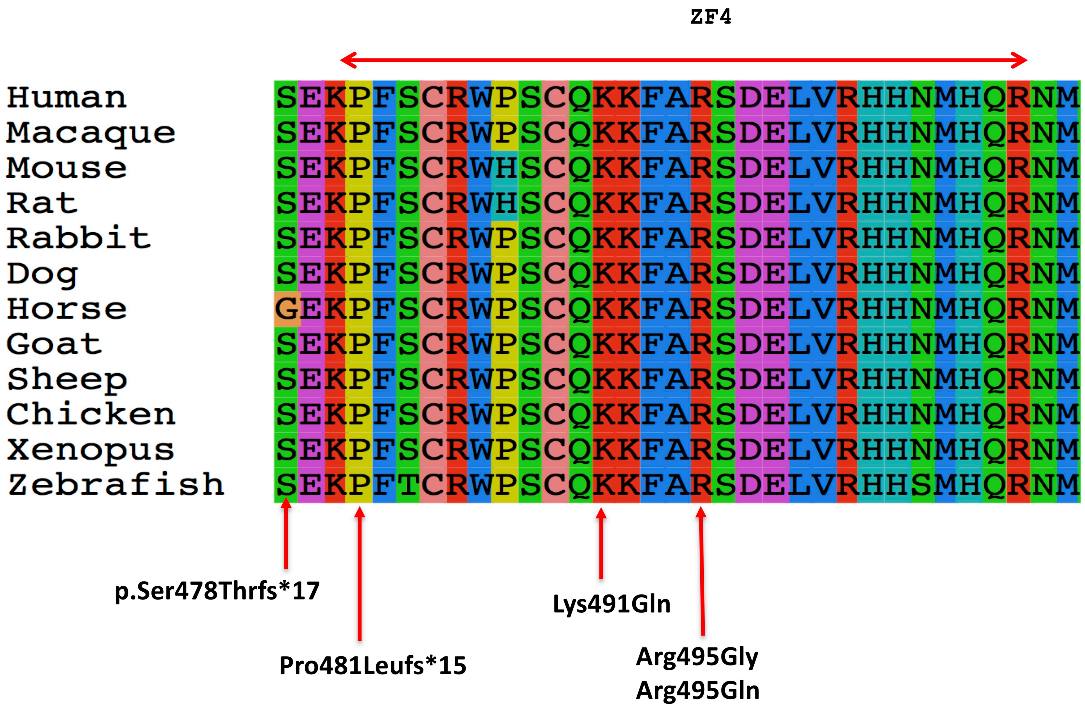


**Figure S2. Evolutionary conservation of ZF4 of WT1 in vertebrate species.**
Protein sequence alignment of the human WT1 ZF4 was performed with that of other vertebrate species. The position of the mutations is indicated by arrows. All the residues are highly conserved in vertebrates.

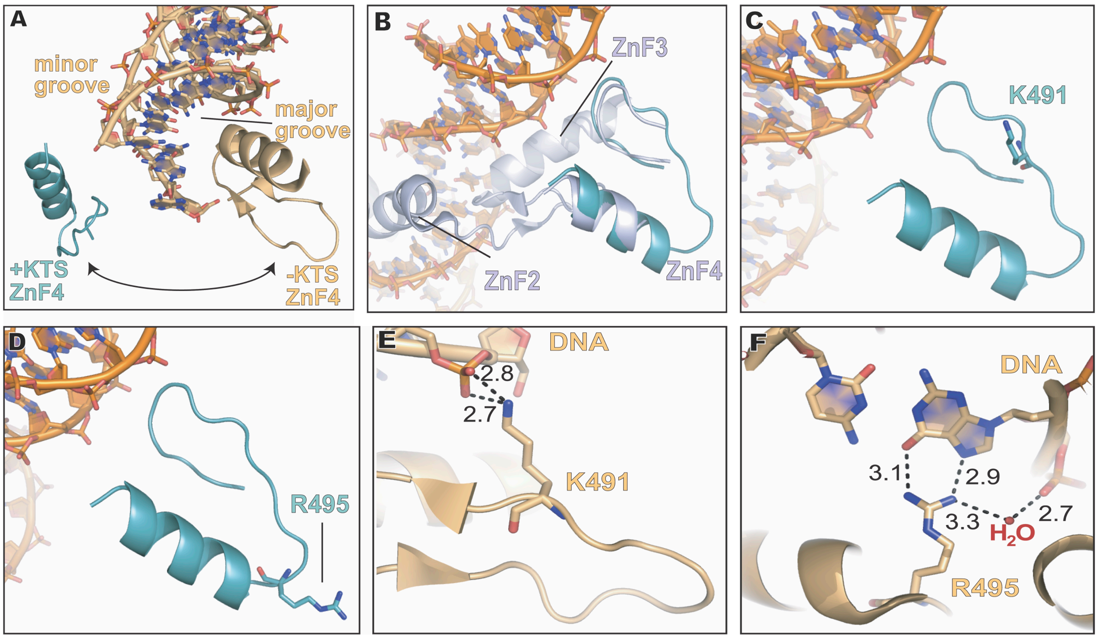


**Figure S3.** ***In silico*** **analysis predicts a disruptive effect of the mutations in the ZF4 of WT1 on DNA binding**

Co-crystal structures of the zinc finger domains of WT1 KTS+ or KTS- sequences (39) were used to generate hypotheses for the molecular impacts of pathogenic variants **(A)** Relative positions of ZF4 in the structure of WT1 KTS+ (aqua) and KTS- (orange) (PDBID 6BLW and 6B0R, respectively) are indicated. **(B)** Superposition of a complete ZF4 (derived from the -KTS structure (aqua)) on the largely-disordered ZF4 of the +KTS structure (grey). **(C and D)** Putative positions of KTS+ Lys491 (**C**) and Arg495 (**D**) with the double-stranded DNA based on the structural alignment from **(B)** **(E) and (F).** Positions of Lys491 (**E**) and Arg495 (**F**) in the WT1 KTS- isoform. Red sphere represents an ordered water molecule. Distances are shown in Ångstrom. The ZF4 in the KTS+ and KTS- isoforms of WT1 binds double-stranded DNA very differently. The KTS+ isoform binds the minor groove, while the KTS- isoform binds the major groove. It has been suggested that ZF4 plays a more substantial role in stabilization of DNA-binding in the KTS- isoform than it does in the KTS+ isoform (39-41). The *in silico* models suggest that mutations of either Arg495 or Lys491, or other mutations that affect ZF4 in KTS+ would impact nucleic acid binding if ZF4 assumes the position observed in the KTS- structure. In any scenario, the fact that two patients presented with complete absence of ZF4 as a result of frameshift mutations indicates that the function of the ZF4 is abolished.

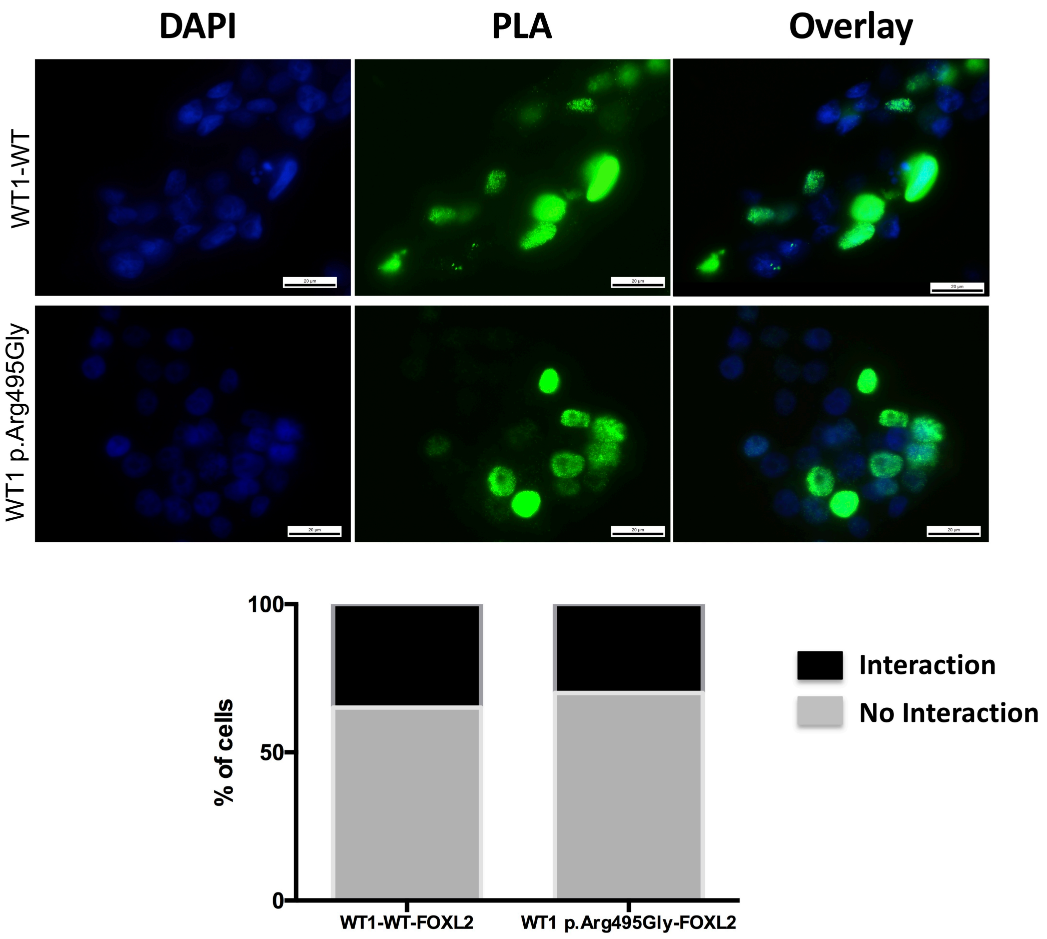


**Figure S4. Protein–protein interaction of WT1 and FOXL2.**
Protein–protein interaction of WT1-wild type and WT1p.Arg495Gly with FOXL2 was analyzed using the Duolink PLA. Nuclei are stained with DAPI (blue) and Duolink signal representing interaction between the proteins is shown in green. Each green dot represents a single dimerization event. Both the wild-type and mutant proteins show similar levels of binding to the FOXL2 protein using the PLA (upper panel). For each condition at least 300 individual cells were counted and the percentage of cells showing interaction were calculated (lower panel).

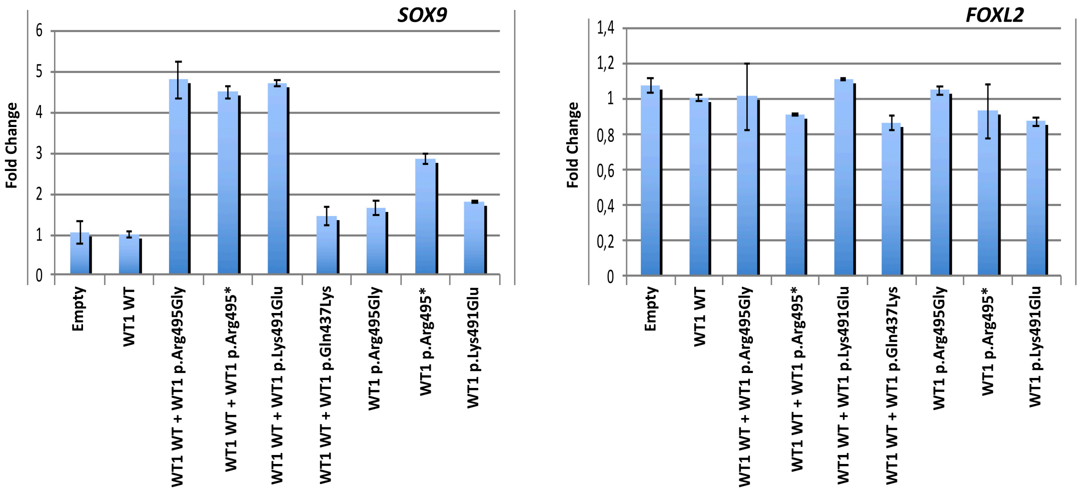


**Figure S5. Endogenous expression of SOX9 and FOXL2 in KGN cells after transfection with plasmids carrying WT1 variants.**

Human granulosa KGN-1 cells were transfected with plasmids encoding mouse WT1-wild-type and variants (WT1p.Arg495Gly, WT1p.Arg495*, WT1p.Lys491Glu and WT1p.Gln437Lys). After transfections, the levels of endogenous Sertoli specific (*SOX9*), and granulosa specific (*FOXL2*) transcripts were measured by qRT-PCR. Data are presented as mean 2-ΔΔCt values, normalized to the housekeeping gene 18S rRNA normalizer gene (RPL19). When transfected together the WT1-wild-type and WT1p.Arg495Gly, WT1p.Arg495* or WT1p.Lys491Glu result in multi-fold induction of endogenous *SOX9* but the same remained unchanged following transfection with a WT1p.Gln437Lys. The levels of endogenous *FOXL2* remain unchanged.

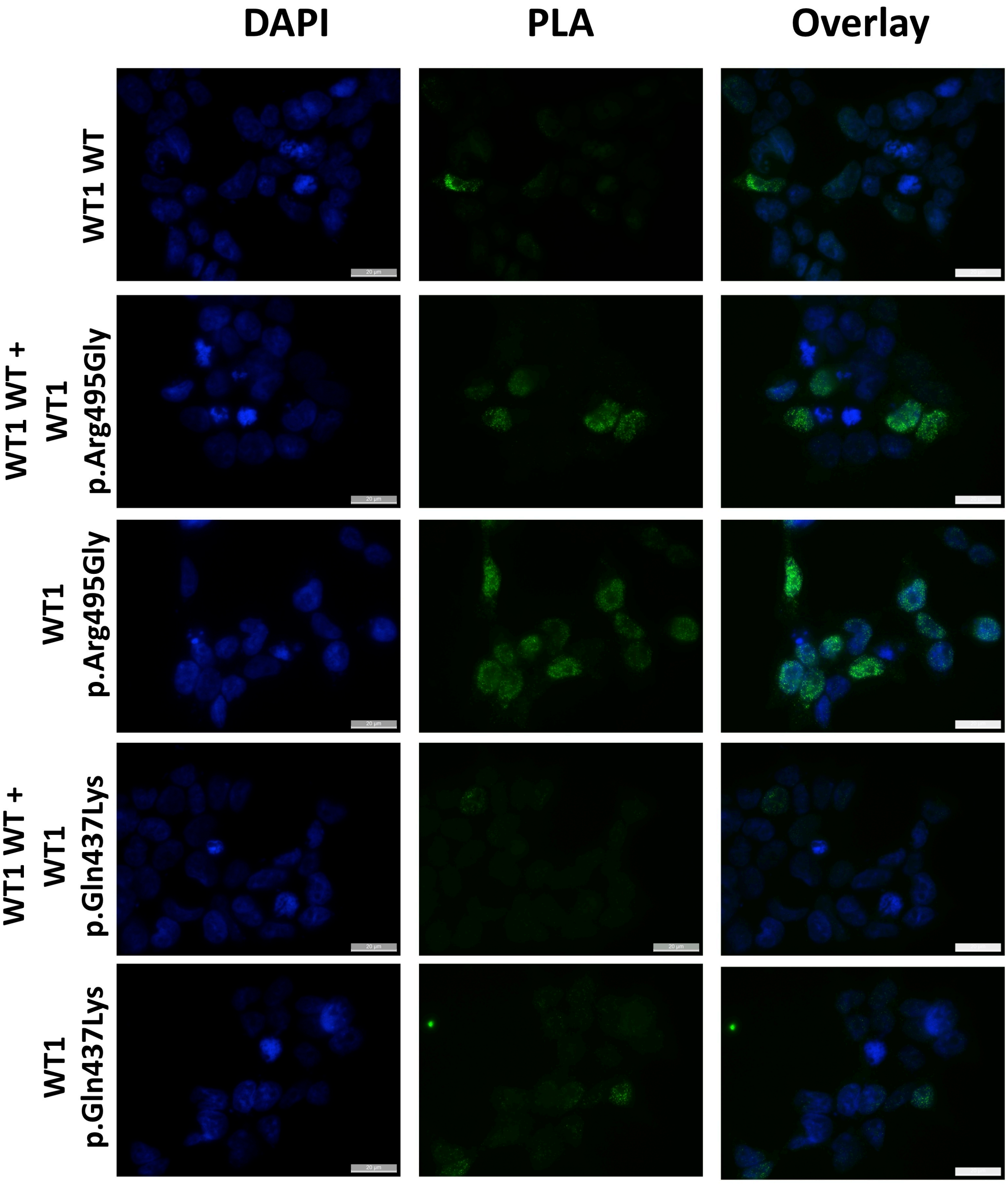
DAPI
PLA
Overlay
WT1 WT
WT1 WT + WT1 p.Arg495Gly
WT1 p.Arg495Gly
WT1 WT + WT1 p.Gln437Lys
WT1 p.Gln437Lys

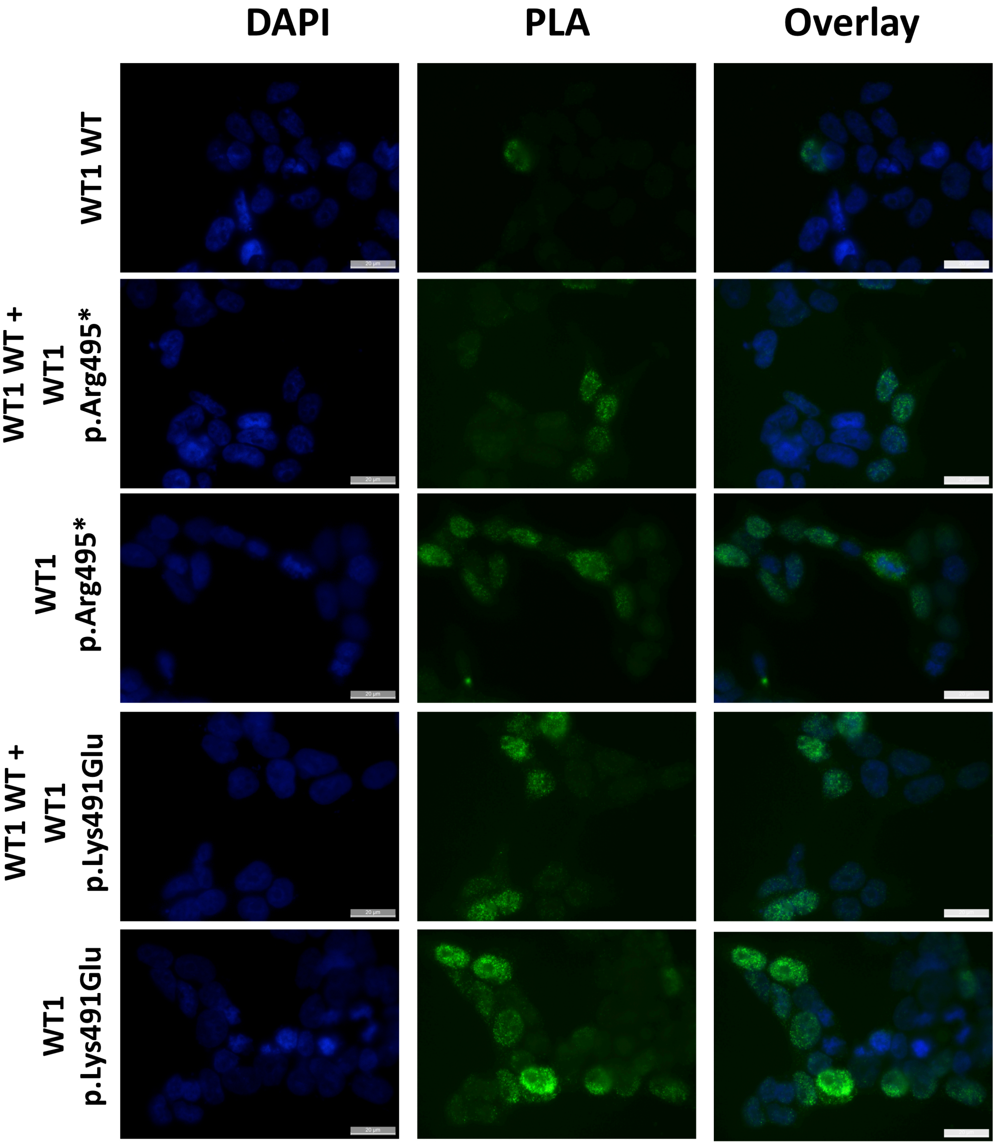
DAPI
PLA
Overlay
WT1 WT
WT1 WT + WT1 p.Arg495*
WT1 p.Arg495*
WT1 WT + WT1 p.Lys491Glu
WT1 p.Lys491Glu

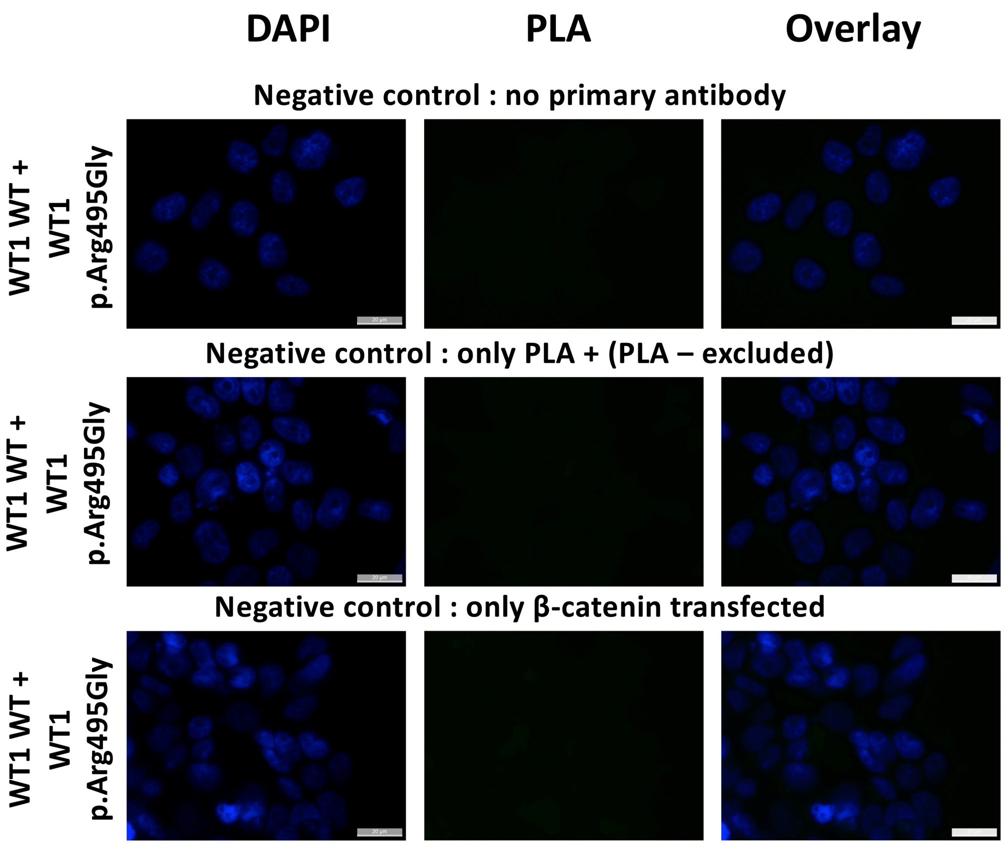
DAPI
PLA
Overlay
Negative control : no primary antibody
WT1 WT + WT1 p.Arg495Gly
Negative control : only PLA + (PLA – excluded)
WT1 WT + WT1 p.Arg495Gly
Negative control : only β-catenin transfected
WT1 WT + WT1 p.Arg495Gly

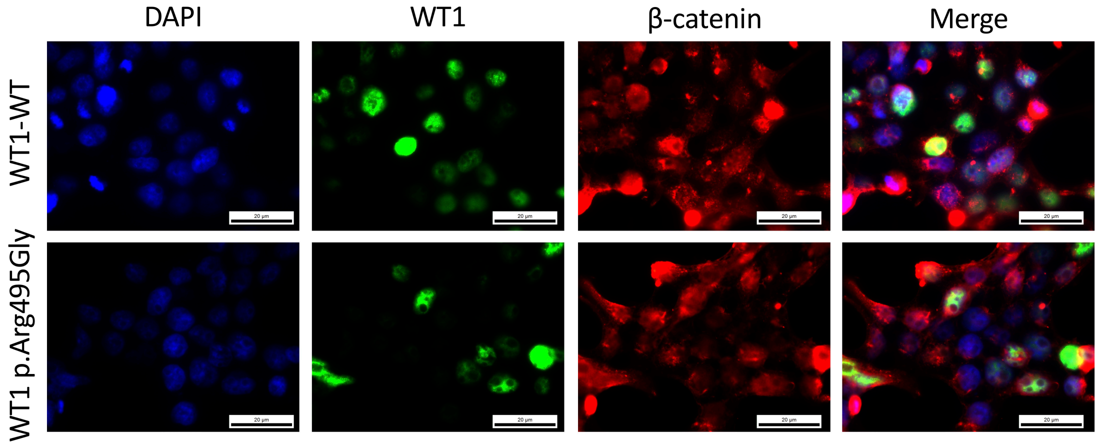
DAPI
WT1
β-catenin
Merge
WT1-WT
WT1 p.Arg495Gly

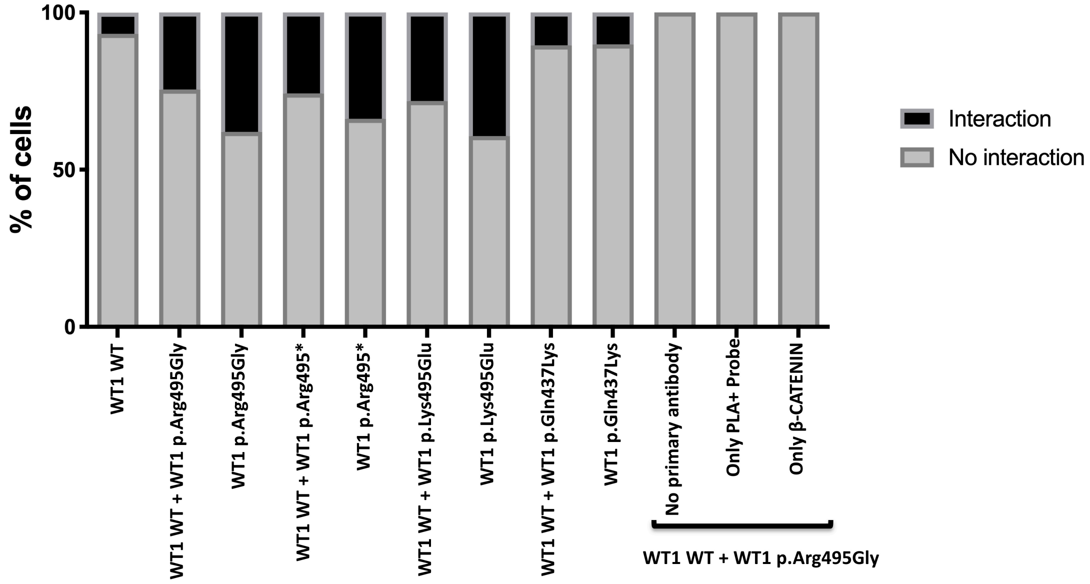


**Figure S6. Protein-Protein Interaction between WT1 and β-CATENIN.**
Plasmids encoding β-CATENIN and WT1-wild-type, WT1p.Arg495Gly, WT1p.Arg495*, WT1p.Lys491Glu or WT1p.Gln437Lys were transiently expressed for 48 h in HEK293-T cells. Protein–protein interaction of WT1-wild-type and variants with β-CATENIN was analyzed using the Duolink PLA. Nuclei are stained with DAPI (blue) and Duolink signal representing interaction between the proteins is shown in green. In contrast to the wild-type, WT1p.Arg495Gly, WT1p.Arg495* and WT1p.Lys491Glu show a significantly increased binding to β-CATENIN protein whereas WT1p.Gln437Lys is comparable to the WT1-wild-type.
The expression of of β-CATENIN and WT1 was assessed in the transfected cells that were analysed for interaction between β-CATENIN and WT1-wild-type and WT1p.Arg495Gly using the Duolink PLA (Fig.4D). As negative control fixed and permeabilized cells were incubated with the indicated primary antibodies or with one of the two PLA probe by pair or alone. Scale bar is 20 µm.
For each condition at least 300 individual cells were counted and the percentage of cells showing interaction were calculated.

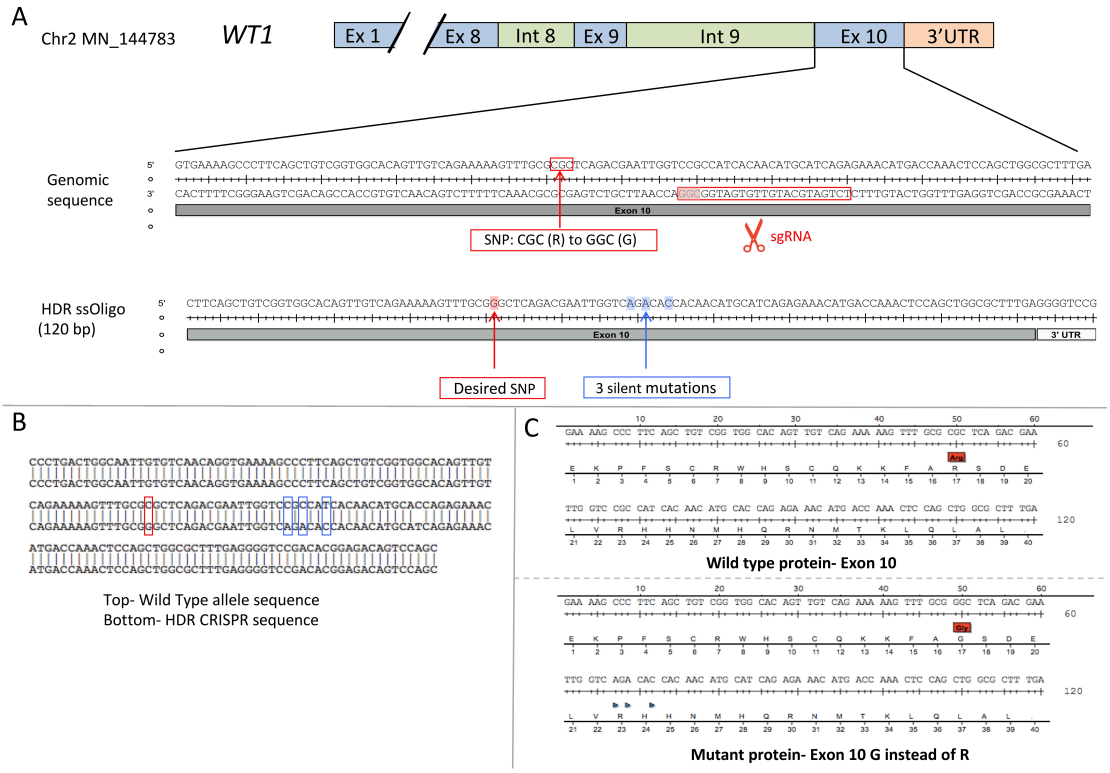


**Figure S7. Using CRISPR/CAS9 to generate the Wt1p.Arg495Gly mutant mouse model.**
(A) A schematic representation of the mouse *Wt1* gene, located on chromosome 2. The mutation was inserted in Exon 10, which encodes for the ZF4. The location of the SNP is indicated by red box and red arrow. In the mouse we wanted to convert a CGC (Arg) into a GGC (Gly), as happened in patient 1. The location of the sgRNA and PAM sequence is indicated with red box and scissors symbol. The sequence of the 120 bp-long ssOligo used to perform homology-dependent-repair (HDR) is presented below the genomic locus. The desired mutation is indicated in red while three silent mutations inserted are indicated in Blue. (B) DNA Blast results of PCR fragment amplified around the mutation site from both wild-type controls and mutant founder. Top line is the wild-type genomic sequence while the bottom line is the mutant founder sequence carrying the HDR allele. The desired mutation is indicated in red while three silent mutations inserted are indicated in blue. (C) Partial translation of Exon 10 from both wild-type allele and HDR allele found in the founder. The Arg at position 17 of exon 10 was changed into a Gly while all other amino acid residues are identical. Blue arrowheads indicate the presence of silent mutations that only altered DNA sequence but not protein residues.

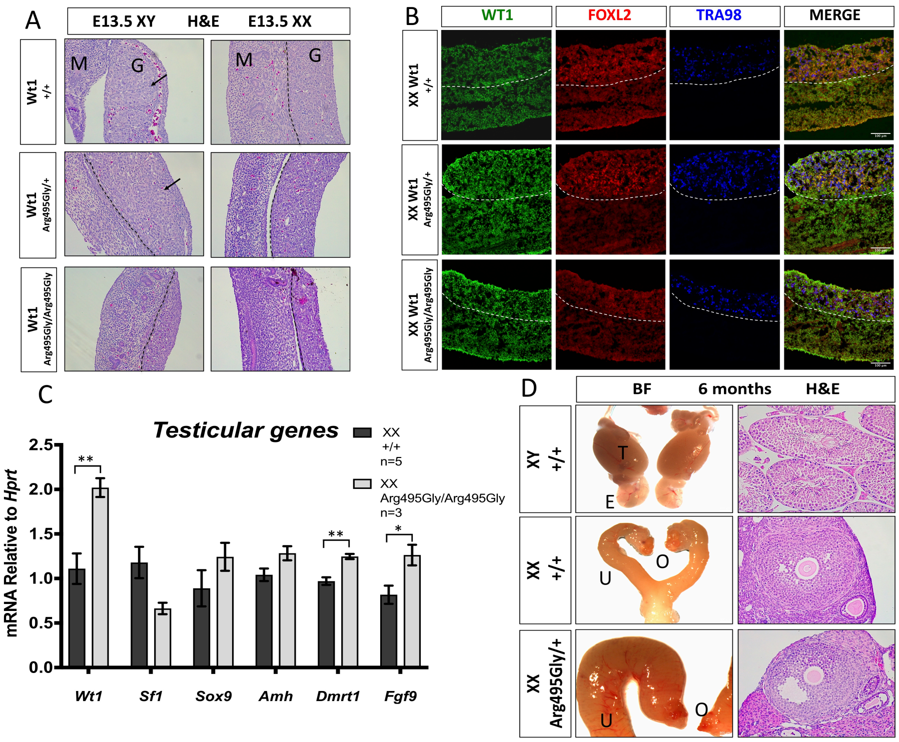


**Figure S8. Extended phenotype of the *Wt1*$^{Arg495Gly}$ mutant mouse.**

(A) Haematoxylin and eosin (H&E) stained sections of wild-type (*Wt1*$^{+/+}$), heterozygous (*Wt1*$^{Arg495Gly/+}$) and homozygous (*Wt1*$^{Arg495Gly/Arg495Gly}$) XY and XX gonads at E13.5. Black arrows indicate the presence of testis cords. Dashed black line marks the border between the gonad and mesonephros. G (Gonad), M (Mesonephros). (B) Immunostaining of E13.5 gonads from XX wild-type, heterozygous and homozygous mice. Gonads were stained for Granulosa-marker FOXL2 (red) and Germ cell-marker TRA98 (Blue). Dashed white line marks the border between the gonad and mesonephros. Scale bars represent 100 µm. (C) Real-time quantitative PCR of genes involved in male sex determination (*Wt1*, *Sf1, Sox9, Amh, Dmrt1,* and *FGF9*) in XX wild type and *Wt1*$^{Arg495Gly/Arg495Gly}$ homozygous gonads at E13.5. Data are presented as mean $2^{-\Delta\Delta Ct}$ values, normalized to the housekeeping gene *Hprt*. Sample size represents the number of individuals and is indicated on the side. Error bars show SEM of $2^{-\Delta\Delta Ct}$ values. P value is presented above the relevant bars (unpaired, two-tailed t-test on $2^{-\Delta\Delta Ct}$ values, *P ≤0.05, **P≤0.01). Dark grey bars: wild-type XX gonads; light grey bars: *Wt1*$^{Arg495Gly/Arg495Gly}$ homozygous XX gonads. (D) Bright field images of gonads and reproductive tract (left panel) and H&E gonadal sections (right panel) of 6 month old wild-type XY, wild-type XX and heterozygous *Wt1*$^{Arg495Gly/+}$ XX mice. Heterozygous XX females have relatively normal looking ovaries but an extremely enlarged uterus. T (Testis), E (Epididymis), O (Ovary), U (Uterus).

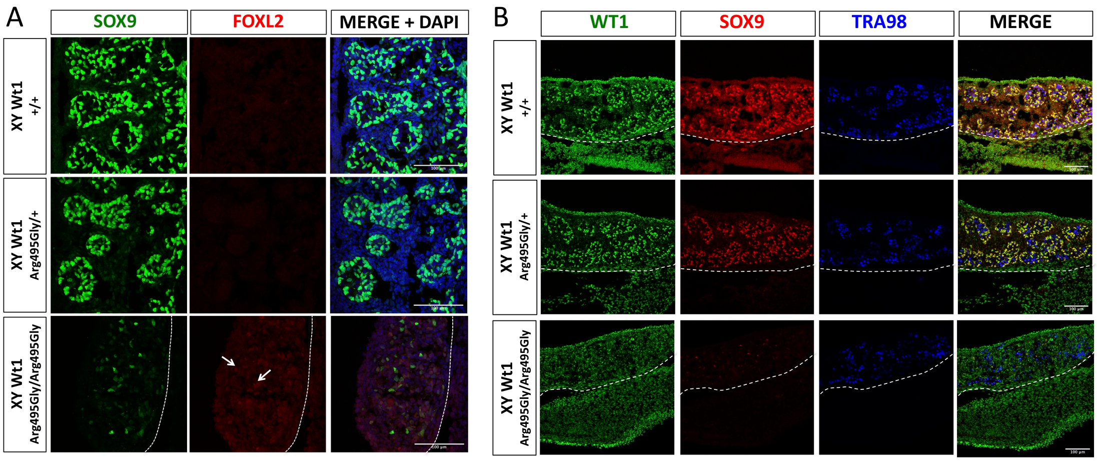


**Figure S9. XY $\textit{WT1}^{Arg495Gly/Arg495Gly}$ homozygous mice are partly sex reversed.** (A) Immunostaining of E13.5 gonads from XY wild-type, heterozygous and homozygous mice. Gonads were stained for Sertoli-marker SOX9 (green), Granulosa-marker FOXL2 (red) and DAPI (blue). Homozygous XY gonads do not present any testis cords and appear with very few scattered SOX9 expressing cells while substantially large amount of FOXL2 positive cells. Dashed white line marks the border between the gonad and mesonephros. (B) Immunostaining of E13.5 gonads from XY wild-type, heterozygous and homozygous mice. Gonads were stained for Sertoli-marker SOX9 (red) and Germ cell-marker TRA98 (blue). Homozygous XY gonads still contain germ cells but those are scattered around instead of being wrapped within testis cords. Scale bars represent 100 µm.

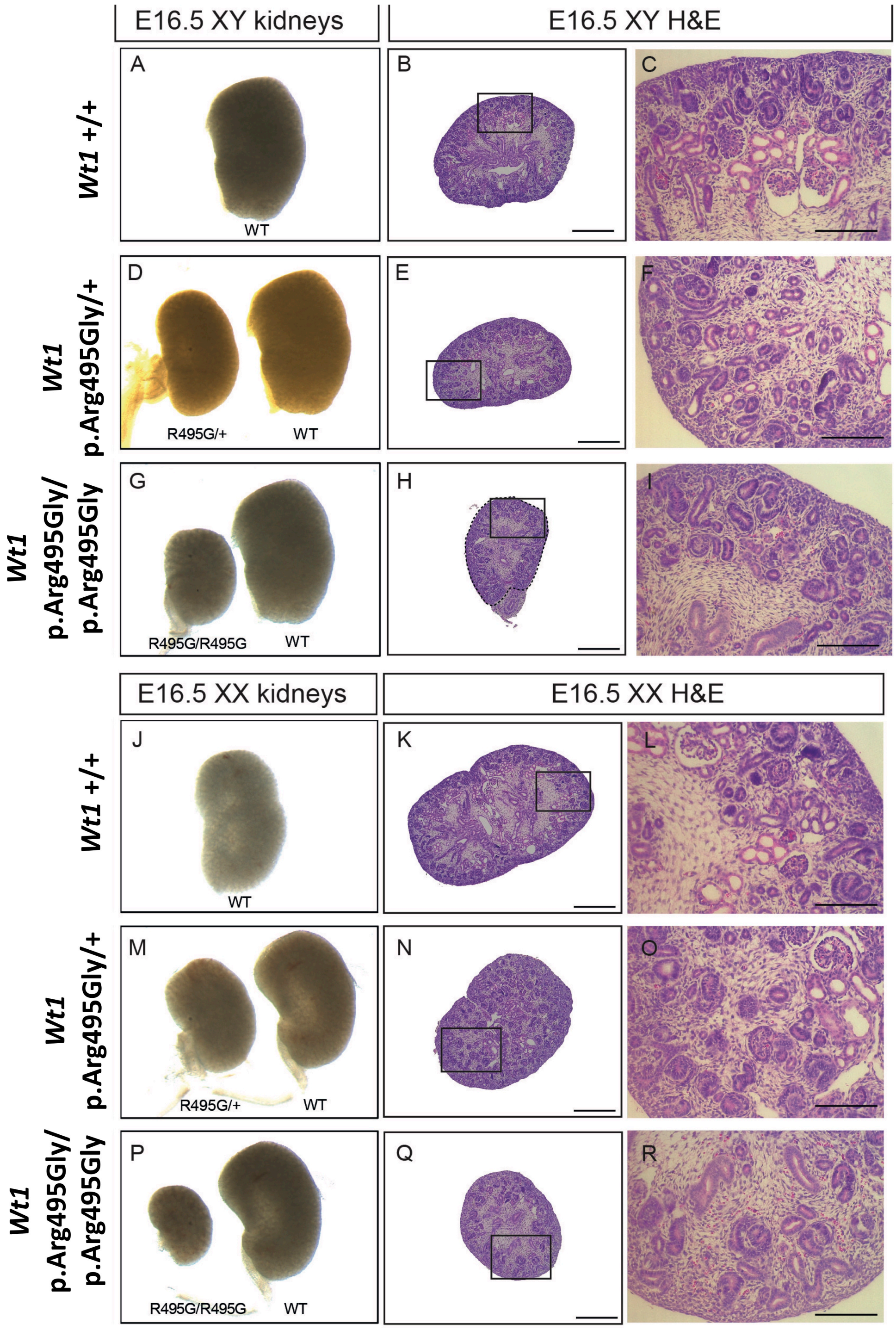


**Figure S10**. **Impaired kidney development in *Wt1*$^{Arg495Gly/Arg495Gly}$ mutant mice.** (**A, D, G; J, M, P**) Brightfield images showing gross morphology of wild-type Wt1 *Wt1*$^{Arg495Gly/+}$, and *Wt1*$^{Arg495Gly/Arg495Gly}$ mutant littermate kidneys at E16.5. Wild-type mice (**A-C, J-K**) show a well-defined nephrogenic zone with several developing glomeruli (outermost layer), a cortex and a medulla (innermost layer). Kidneys of *Wt1*$^{Arg495Gly/+}$ (D-F, M-N) and *Wt1*$^{Arg495Gly/Arg495Gly}$ mutants (G-I, P-R) appear considerably smaller than wild-type controls. **(B, E, H)** H&E staining of sagittal sections of wild-type, *Wt1*$^{Arg495Gly/+}$ and *Wt1*$^{Arg495Gly/Arg495Gly}$ kidneys. (Scale bar 500 µm). **(C, F, I)** Higher magnification of boxed areas in B, E and H. (Scale bar 150 µm).

Thickness of both cortex and medulla is reduced in *Wt1*$^{Arg495Gly/Arg495Gly}$ kidneys compared to wild-type.

E16.5 XY and XX Wild-Type Kidney: These sections exhibit a moderately mature kidney containing well-developed renal corpuscles (Malpighian corpuscle) with a defined capsule of Bowman and investing a glomerular tuft. Glomerular tufts are surrounded by an adequate urinary/glomerular space and the urinary and vascular poles are also discernible. The macula densa is frequently observed. Although incompletely developed and immature, proximal and distal convoluted tubules are morphologically distinguishable. The outer cortex and juxtamedullary cortex are discernible. The corticomedullary junction is not sharply demarcated and the pelvis is discernible. Mesenchymal cells are abundant.

E16.5 XY and XX heterozygous (*Wt1*$^{Arg495Gly/+}$) kidney: No histological differences are observed in comparison with the wild-type kidney.

E16.5 XY and XX homozygous (*Wt1*$^{Arg495Gly/Arg495Gly}$) kidney: Age-matched kidneys are significantly smaller. The cortex is architecturally homogeneous with no clear boundary between the outer cortex and juxtamedullary cortex. There is a significant lack of developed renal corpuscles and glomerular spaces are not observed. Furthermore, the number of renal corpuscles is subjectively lower than in the wild-type and *Wt1*$^{Arg495Gly/+}$ kidneys. Similarly, tubules are more immature in comparison with the wild-type and *Wt1*$^{Arg495Gly/+}$ kidneys. Morphological differences indicating differentiation towards proximal or distal convoluted tubules are not observed. Mesenchymal cells are abundant. Microscopic examination reveals significant morphological differences with wild-type and *Wt1*$^{Arg495Gly/+}$ kidneys. Although histologically normal, and considering that, as per history, they are age-matched organs, E16.5 *Wt1*$^{Arg495Gly/Arg495Gly}$ kidneys do not contain developed glomerular corpuscles and tubules are underdeveloped. The low number and small size of both renal corpuscles and tubules account for the relative smaller size of the examined kidneys in comparison with *Wt1*$^{Arg495Gly/+}$ and wild-type. Otherwise there is no evidence of degeneration or inflammation in the examined sections.

**References**


39. Wang D*, et al.* (2018) Role for first zinc finger of WT1 in DNA sequence specificity: Denys-Drash syndrome-associated WT1 mutant in ZF1 enhances affinity for a subset of WT1 binding sites. *Nucleic Acids Res* 46(8):3864-3877.
40. Kabsch W, Kabsch H, & Eisenberg D (1976) Packing in a new crystalline form of glutamine synthetase from Escherichia coli. *J Mol Biol* 100(3):283-291.
41. Winn MD*, et al.* (2011) Overview of the CCP4 suite and current developments. *Acta Crystallogr D Biol Crystallogr* 67(Pt 4):235-242.
42. Lourenco D*, et al.* (2011) Loss-of-function mutation in GATA4 causes anomalies of human testicular development. *Proc Natl Acad Sci U S A* 108(4):1597-1602.
43. Bashamboo A*, et al.* (2014) Mutations in the FOG2/ZFPM2 gene are associated with anomalies of human testis determination. *Hum Mol Genet* 23(14):3657-3665.
44. Portnoi MF*, et al.* (2018) Mutations involving the SRY-related gene SOX8 are associated with a spectrum of human reproductive anomalies. *Hum Mol Genet* 27(7):1228-1240.
45. Pannetier M, Renault L, Jolivet G, Cotinot C, & Pailhoux E (2005) Ovarian-specific expression of a new gene regulated by the goat PIS region and transcribed by a FOXL2 bidirectional promoter. *Genomics* 85(6):715-726.
46. Evtouchenko L, Studer L, Spenger C, Dreher E, & Seiler RW (1996) A mathematical model for the estimation of human embryonic and fetal age. *Cell Transplant* 5(4):453-464.
47. Jorgensen A*, et al.* (2015) Ex vivo culture of human fetal gonads: manipulation of meiosis signalling by retinoic acid treatment disrupts testis development. *Hum Reprod* 30(10):2351-2363.
48. Mitchell RT*, et al.* (2008) Germ cell differentiation in the marmoset (Callithrix jacchus) during fetal and neonatal life closely parallels that in the human. *Hum Reprod* 23(12):2755-2765.
49. Gonen N, Quinn A, O'Neill HC, Koopman P, & Lovell-Badge R (2017) Normal Levels of Sox9 Expression in the Developing Mouse Testis Depend on the TES/TESCO Enhancer, but This Does Not Act Alone. *PLoS Genet* 13(1):e1006520.
50. Beverdam A & Koopman P (2006) Expression profiling of purified mouse gonadal somatic cells during the critical time window of sex determination reveals novel candidate genes for human sexual dysgenesis syndromes. *Hum Mol Genet* 15(3):417-431.
